# A novel differential evolution algorithm with multi-population and elites regeneration

**DOI:** 10.1371/journal.pone.0302207

**Published:** 2024-04-25

**Authors:** Yang Cao, Jingzheng Luan

**Affiliations:** 1 School of Computer Science and Engineering, Shenyang Jianzhu University, Shenyang, China; 2 Liaoning Province Big Data Management and Analysis Laboratory of Urban Construction, Shenyang, China; 3 Shenyang Branch of National Special Computer Engineering Technology Research Center, Shenyang, China; Gulf University for Science and Technology, KUWAIT

## Abstract

Differential Evolution (DE) is widely recognized as a highly effective evolutionary algorithm for global optimization. It has proven its efficacy in tackling diverse problems across various fields and real-world applications. DE boasts several advantages, such as ease of implementation, reliability, speed, and adaptability. However, DE does have certain limitations, such as suboptimal solution exploitation and challenging parameter tuning. To address these challenges, this research paper introduces a novel algorithm called Enhanced Binary JADE (EBJADE), which combines differential evolution with multi-population and elites regeneration. The primary innovation of this paper lies in the introduction of strategy with enhanced exploitation capabilities. This strategy is based on utilizing the sorting of three vectors from the current generation to perturb the target vector. By introducing directional differences, guiding the search towards improved solutions. Additionally, this study adopts a multi-population method with a rewarding subpopulation to dynamically adjust the allocation of two different mutation strategies. Finally, the paper incorporates the sampling concept of elite individuals from the Estimation of Distribution Algorithm (EDA) to regenerate new solutions through the selection process in DE. Experimental results, using the CEC2014 benchmark tests, demonstrate the strong competitiveness and superior performance of the proposed algorithm.

## Introduction

Differential Evolution (DE) is a powerful search method proposed by Storn and Price [1,2]. This simple yet efficient algorithm has been widely applied and has shown remarkable effectiveness in solving optimization problems across various fields and real-world applications [3–7]. In a manner akin to other Evolutionary algorithms (EAs), DE gradually approaches the global optimal solution in each generation through mutation, crossover, and selection operations. In addition, DE is one of the most effective EAs currently in use [8]. Moreover, DE offers flexibility and adaptability, allowing researchers and practitioners to customize its parameters and strategies according to specific problem characteristics. Therefore, some researchers have also combined DE with other metaheuristic algorithms to improve performance by leveraging the strengths of each algorithm [9]. This adaptability makes DE well-suited for a wide range of optimization problems, spanning from continuous to discrete domains.

In DE, the individuals within the population are referred to as target vectors. Through the process of mutation, a mutant vector is created by introducing perturbations to a target vector uses the difference vectors of other individuals within the population. Subsequently, the crossover operation combines the parameters of the mutant vector with those of a parent vector chosen from the population, resulting in the generation of a trial vector. The process of determining the next generation involves evaluating the fitness values of the trial vectors and their corresponding parent vectors. This is done through a selection operation that engages in a one-to-one competition [10,11]. The efficacy of DE heavily relies on the chosen mutation strategy and crossover operator. In addition, the intrinsic control parameters, specifically the population size (*NP*), scaling factor (*F*), and crossover rate (*Cr*), act as crucial factors in striking a balance between population diversity and algorithm convergence speed.

DE possesses numerous benefits, including ease of implementation, dependability, speed, and adaptability [3]. In general, DE is known for its good global exploration capabilities and its ability to approach the global optimum in optimization problems. However, its exploitation rate, which refers to the ability to exploit local search spaces efficiently, is relatively slow compared to other algorithms [12]. Furthermore, the parameters of DE are problem-related, making it difficult to adapt for different problems. Additionally, the performance of DE tends to worsen as the dimension of the search space increases[13].

The distribution estimation algorithm (EDAs) [14–16] is a recent branch that has emerged. EDAs are random optimization method that extracts samples from the probability distribution of each generation’s expected solution estimation. An important feature of EDA and other EAS is the establishment of a probabilistic model based on elite individuals sampled from the population. This model-based optimization approach enables EDAs to solve many of the complex problems Hauschild and Pelikan [17]. From the literature [18–20], these modifications and improvements to DE mainly focus on developing new mutation rules, and there are some attempts to adjust control parameters in an adaptive or adaptive manner, as well as the exploitation and utilization of elite solutions.

The performance of an optimal optimization algorithm depends on its ability to dynamically adjust the ability to avoid falling into local optima and provide larger steps. during different stages of evolution. When it comes to enhancing search strategies, a well-designed search strategy should promote initial exploration and enable individuals to leverage the information obtained from the search space to enhance and optimize the solution.

The integration of JADE [21] and the newly proposed mutation strategy was implemented, resulting in a new DE variant called EBJADE, which enhanced their search ability for difficult and complex optimization problems.Therefore, this paper has three main innovative aspects:

1. Introducing a new DE algorithm, it introduces a mutation strategy for enhancing exploitation capabilities known as DE/current-to-ord/1 (abbreviated as ord). To perform mutation, this approach utilizes the global vectors and applies a sorting technique. For each objective vector, the method chooses one vector from the top *p* best vectors, from the *p* vectors in the median rank, this paper selects one vector as the median vector, and from the bottom *p* worst vectors, this paper selects one vector as the worst vector.
2. The entire population is divided into two indicator subpopulations and one reward subpopulation. Both variants of DE are combined, and the reward subpopulation is dynamically allocated based on the historical performance of each variant, favoring the one with better historical performance.
3. Utilizing the concept of sampling from the Elite section of EDAs. After the selection process in DE, in this paper, the location of the elite individual is sampled by using the neighborhood area of the elite solution. Inspired by the above, new individuals approach the elite solutions in the probability model are sampled, enabling us to further exploit the promising regions discovered so far and potentially find better solutions. Consequently, this leads to the generation of more competitive solutions, which serve as guides for evolution and enhance the chances of DE avoiding local optima.

The remaining sections of this paper are organized as follows:Section 2 introduces the original DE algorithm, including its typical mutation operator, crossover operator, and selection operator. Section 3 provides a review of related work. Next, in Section 4 proposes various multi-population strategies, mutation strategies, and elitist solution adjustments. Section 5 reports and discusses the effectiveness of the proposed algorithm based on computational results and comparisons with other advanced algorithms using the CEC2014 benchmark. The experimental results demonstrate that the proposed algorithm exhibits strong competitiveness in terms of the robustness, stability, and quality of the obtained solutions. Finally, Section 6 draws conclusions based on the findings.

## Original differential evolution algorithm

In this section, this paper describes the fundamental process of Differential Evolution (DE) and introduce the necessary symbols and terminology which aid in explaining the algorithm proposed subsequently.

The original DE algorithm is a type of evolutionary algorithm used for continuous optimization problems. It follows a series of three main steps (mutation, crossover, and selection) in an iterative manner until a predefined termination criterion is satisfied. This research starts by forming an initial population called *P*, which is comprised of *NP* individuals that are randomly selected. Every individual in the population is represented as a vector *X*_*i*_, where *X*_*i*_ = {*x*_*1*,*i*_,*x*_*2*,*i*_,*x*_*3*,*i*_,…*x*_*D*,*i*_}, with *i* ranging from 1 to *NP*. In this context, *i* denotes the total number of individuals, and D represents the dimensionality of the solution space. Since the population evolves during the process, this paper introduces the concept of generation time, denoted as *G*, with values ranging from 0 to *G*_*max*_, where G_max_ indicates the maximum number of iterations. For the *i*th individual in the *G*th generation, this paper represents it as *X*_*i*,*G*_,*X*_*i*,*G*_ = {*x*_*1*,*i*,*G*_,*x*_*2*,*i*,*G*_,…*x*_*D*,*i*,*G*_}. The lower and upper boundaries for each dimension in the search space are denoted as *X*_*l*_ and *Xu*, respectively. Specifically, *X*_*l*_ = {*x*_*1*,*l*_,*x*_*2*,*l*_,…*x*_*D*,*l*_} and *X*_*u*_ = {*x*_*1*,*u*_,*x*_*2*,*u*_,…*x*_*D*,*u*_}. This paper initializes the population *P*_*0*_ by randomly generating individuals within the specified bounds using a uniform distribution. Subsequently, the DE operators mutation, and crossover, are used to evolve these individuals and generate trial vectors. These trial vectors are then compared with their corresponding parents, determining which vectors should retained for the next generation. The overall steps of DE are described in detail as follows:

### Initialization

To initiate the optimization process, this paper quires the generation of an initial population, denoted as *P*_*0*_. Usually, the value of each component (indexed by *j*) of the *i*th individual (indexed by *i*) in the population is generated using the following formula:

$$x_{j,i}=x_{j,l}+rand\times (x_{j,u}-x_{j,l})$$ (1)

In the formula, the "*rand*" function generates a random number from a uniform distribution where the values are within the range of [0,1].

### Mutation

In generation *G*, for each target vector *X*_*i*,*G*_, a mutation vector *V*_*i*,*G*_ is generated using the following process:

$$V_{i,G}=X_{r1,G}+F\times (X_{r2,G}-X_{r3,G}),r1\neq r2\neq r3\neq i$$ (2)

This paper randomly selects three indices *r1*, *r2*, and *r3* from the set {1,2,3,…,*NP*}, where *NP* represents the population size. The real number *F* is used to control the amplification of the vector difference (*X*_*r2*,*G*_*-X*_*r3*,*G*_).If any component of the mutation vector exceeds the boundaries of the search space, this study employs an equation to generate a replacement value for that component. This approach is known as DE/rand/1/bin, which is a commonly used mutation strategy. There are also other commonly used mutation strategies, including:

DE/best/1/bin

$$V_{i,G}=X_{best,G}+F\times (X_{r1,G}-X_{r2,G})$$ (3)

DE/current-to-best/1/bin

$$V_{i,G}=X_{i,G}+F\times (X_{best,G}-X_{r1,G})+F\times (X_{r2,G}-X_{r3,G})$$ (4)

Within the DE/rand/1/bin mutation strategy context, the selection of these indices is conducted from the set (1, 2,…, *NP*), with *NP* denoting the size of the population. It is important to note that these indices should be different from the index *i*, which refers to the current target vector.This scaling operation amplifies the impact of the difference vector on the mutation process. The specific value of *F* determines the extent to which the difference vector influences the mutant vector. By adjusting the value of *F*, the algorithm is capable of achieving a trade-off between exploration and exploitation within the search space.

### Crossover

In DE, there are two main types of crossover: binomial and exponential. Here, this paper will provide a detailed explanation of binomial crossover. In binomial crossover, the target vector *X*_*i*,*G*_ is combined with the mutation vector *V*_*i*,*G*_ to generate the trial vector *U*_*i*,*G*_.This crossover is achieved using the following scheme:

$$u_{i,j,G+1}=\left\{ \begin{array}{c} v_{i,j,G}\;if\;{rand}_{j}\leq Cr\;or\;j=j_{rand}\\ x_{i,j,G}\;otherwise \end{array}\right.$$ (5)

Where *rand* is a uniformly distributed random number in the range [0,1], *Cr* ∈[0,1] is called the crossover rate, controlling how many components are inherited from the mutation vector, and *jrand* is a uniformly distributed random integer in the range [1, *D*]. It ensures that at least one component of the trial vector is inherited from the mutation vector.

### Selection

In the process of selection operation, the target vector *X*_*i*,*G*_ is compared with the trial vector *U*_*i*,*G*_, and the vector with better fitness value is entered into the next generation.


$$X_{i,G+1}=\left\{ \begin{array}{l} U_{i,G},\;f(U_{i,G})\leq f(X_{i,G})\\ \;X_{i,G},\;otherwise \end{array}\right.$$ (6)

## Related work

In fact, the performance of the Differential Evolution (DE) algorithm primarily depends on the selected mutation/crossover strategy and the associated control parameters. Due to the limitations of DE mentioned earlier in the introduction, many researchers have been devoted to improving DE. Consequently, several new techniques have been proposed by researchers to overcome its shortcomings and enhance its performance. This section will provide a brief overview of these techniques.

First, improving different adaptive parameters and multi mutation strategies has attracted many researchers. Qin and Suganthan [22] and Qin et al. [23] introduced a modification of DE called self-adaptive differential evolution (SaDE). In SaDE, various strategies are incorporated, and their influence in the search procedure is adjusted by considering their past success rates. Brest et al. [24] suggested an adaptive approach called jDE, in which the control parameters *F* and *Cr* are encoded within individuals and dynamically adjusted throughout the operation of DE. Zhang and Sanderson [21] introduced a novel DE algorithm known as JADE, which enhances the optimization performance by integrating an optional external archive, employing a new mutation strategy called DE/current-to-pbest, and adaptively updating control parameters. Through simulation results, it has been demonstrated that JADE surpasses or is on par with other classical or adaptive DE algorithms in terms of convergence performance. Gong et al. [25] to adaptively select more suitable strategies for specific problems and further improve the performance of DE, a simple strategy adaptation mechanism (SaM) has been implemented. By combining SaM with JADE [21,26], the SaJADE algorithm is proposed. Tanabe and Fukunaga [27] presented an enhanced version of the JADE algorithm called Success History based DE (SHADE). Unlike JADE, SHADE does not sample *F* and *Cr* values from a gradually adapting probability distribution. Instead, it utilizes a historical memory archive, *MCr* and *MF*. The historical memory archive stores a collection of Cr and F values that have exhibited good performance in recent iterations. R. Tanabe and A. Fukunaga [28] using Linear Population Size Reduction (L-SHADE) to improve the search performance of SHADE, which won the championship in the CEC2014 benchmark function test. Ali W et al. [12] introduces two novel mutation strategies. This enhancement boosted both the global and local search capabilities while enhancing the speed of convergence. Meng et al. [29] proposed a mutation strategy based on the historical population, which is known as the novel DE variant Hip-DE. This mutation strategy incorporates information from the historical population, which is essentially a collection of discarded *X*_*i*_ during the selection phase, with a size of 5*15*D. Xia et al. [30] proposed an algorithm called NFDDE, which introduces novelty as a driving force to address the limitations of fitness-based driving forces in the algorithm. The average Euclidean distance between similar individuals is used to calculate their individual novelty, and the weight between the two driving forces is adjusted using a proportion factor *p*. The adaptive adjustment of *F* and *Cr* references the history-based approach in SHADE.

On the flip side, the utilization of multiple populations strategy is widely acknowledged as an effective technique to foster exploration without compromising the diversity within the population. Thus, Wang et al. [31] proposed a new adaptive multi-population DE algorithm (AMPDE). It utilizes multi-population, with each subpopulation being assigned a crossover operator and disturbed based on this crossover operator. The comprehensive adjustment of subpopulation sizes is based on the relative contributions of each subpopulation to the acquired external archive. The two-stage local search helps to improve the diversity of the external archive and the quality of solutions. Wu et al. [32] proposed that EDEV is not just a collection of mutation strategies, but rather a high-level set comprising multiple DE variants. This set includes JADE [22], EPSDE [33], and CoDE [34]. The population of EDEV is divided dynamically into several subpopulations, which include three indicator subpopulations and one reward subpopulation. Each of the smaller indicator subpopulations is associated with a specific mutation strategy, whereas the larger reward subpopulation is allocated as an additional reward to the mutation strategy that is currently performing the best. In this way, dynamic allocation of computational resources among mutation strategies is realized, and it is expected that the optimal mutation strategy will receive the most computational resources. Mallipeddi et al. [33] developed a DE algorithm with mutation strategy and a set of parameter values (EPSDE). In EPSDE, a set of different mutation strategies, along with their respective control parameter values, coexist and compete to produce offspring throughout the entire evolution process. A novel approach named Composite Differential Evolution (CoDE) was introduced by Wang et al. [34]. This method employs three distinct mutation strategies and three sets of control parameters. The mutation strategies and different control parameters are randomly combined to generate experimental vectors. Li et al. [35] proposed a new method, which has been developed to replace the grouping method in MPEDE, and the new grouping method utilizes policy sorting to allocate computing resources to different policies.

In the past two decades since the initial proposal of the basic DE algorithm, numerous variants have been developed. Nonetheless, there has been limited research dedicated to the exploration of the neighborhood surrounding elite solutions. This unexplored territory holds great promise, as it aligns with the principle of optimality. Deng et al. [36] introduced the Elite-Regeneration (ERG) framework, which defines the elite population as a collection of individuals that exhibit high fitness values. Through the selection process in DE, these elites are regenerated. The regeneration process includes generating a new individual from the search space surrounding each elite individual. This new individual is created by sampling from probability models such as Gaussian or Cauchy distributions. Inspired by this, the EBJADE proposed in this paper combines the multi group multi strategy mechanism with the elite solution regeneration mechanism. Tian et al. [37] proposed a novel numerical optimization algorithm based on DE, which aims to balance exploration and exploitation by leveraging neighborhood information. To efficiently address the search needs of each individual, the algorithm constructs a set of elite individuals using a ring topology. It then adaptively selects appropriate elite individuals based on their performance within their neighborhoods. This selection process serves to guide the search. Based on the ideas of DE/EDA, Dong et al. [38] proposes another method that combines distribution estimation algorithms with differential evolution for global optimization. A hybrid differential evolution and distribution estimation algorithm based on neighborhood search is proposed [39], combining the advantages of distribution estimation algorithm and differential evolution algorithm. At the same time, two mutation operators are used to enhance the search ability of the algorithm, and a chaotic strategy is introduced to update the parameters of DE. Fan et al. [40] used alternative probability models in sampling to improve population diversity. And then combining the algorithm with DE and adopting an adaptive strategy to improve the convergence speed of the algorithm. In order to fully utilize the powerful development of DE and the powerful exploration of EDA, this paper proposes an improved differential evolution algorithm IDE-EDA for mixed distribution estimation algorithms [41].

Indeed, the main changes and advancements in Differential Evolution (DE) have primarily revolved around adaptive control parameter adjustment. Nevertheless, there have been additional enhancements made by modifying the structure and mechanisms of the core DE algorithm, as well as introducing new mutation rules. These modifications aim to enhance DE’s ability to perform local and global searches and address challenges such as stagnation or premature convergence. Research on elite solution neighborhoods has demonstrated the potential of these neighborhoods in generating superior solutions.

## Propose algorithm

In this section, divide it into three parts. The first part introduces two mutation strategies, namely the novel mutation strategy proposed in this paper, DE/current to pbest/1 with external archive, and parameter adaptive mechanism. Then the second part introduces multiple populations strategies. Finally, we used the sampling method used in elites regeneration to sample the candidate individuals around the elite solution.

### Two search strategies

The two search strategies have different characteristics, one with strong exploration ability and the other with strong exploitation ability. Each subpopulation has different search strategies to to provide the ability to avoid falling into local optima and provide larger steps.

1. DE/current-to-ord/1 (abbreviated as ord)

In order to overcome the limitations of the JADE strategy, which exhibits fast but less reliable convergence performance, a more refined search strategy has been introduced. This strategy is more greedy and less exploratory, with a clearer directionality that moves closer to the direction of the best solution in the current population and away from the direction of the worst solution in the current population.


$$V_{i,G+1}=X_{i,G}+F_{i}\times (X_{ptbest,G}-X_{i,G})+F_{i}\times (X_{ptmedian,G}-X_{ptworst,G})$$ (7)

Where, *X*_*i*,*G*_ represents the individual in the *G*th generation, *X*_*ptbest*,*G*_ represents the top *pt*% elite individuals in the population in the *G*th generation, *X*_*ptmedian*,*G*_ represents the middle *pt*% individuals in terms of fitness in the population in the *G*th generation, and *X*_*ptworst*,*G*_ represents the bottom *pt*% individuals in terms of fitness in the population in the *G*th generation.

In the mutation equation, this paper observes that by incorporating a target function value with sorting in this mutation strategy, the objective vector is not consistently pulled towards the identical optimal position identified by the entire population. When local optima are present, this increases the possibility of escaping from local optima. Moreover, the second perturbation part of the mutation is formed by the difference vector in the direction of a randomly selected vector from the top P vectors with better fitness values that have been pre-sorted. Hence, the directed perturbation in the proposed mutation can be analogized to the notion of a gradient, as the difference vector aligns from the inferior vector towards the superior vectors. Due to the fact that the difference vector is directed from the worst vector to the better vector [42]. In contrast to the DE/current-to-pbest/1 strategy, this approach consistently adopts the direction of the best vector for all vectors, while also moving opposite to the direction of the worst vector. This strategy facilitates the algorithm in exploring various sub-regions around the objective vector within the search space during the optimization process. As a result, this optimization process, it has the potential to achieve enhanced performance on specific problem instances.

**Fig 1:**
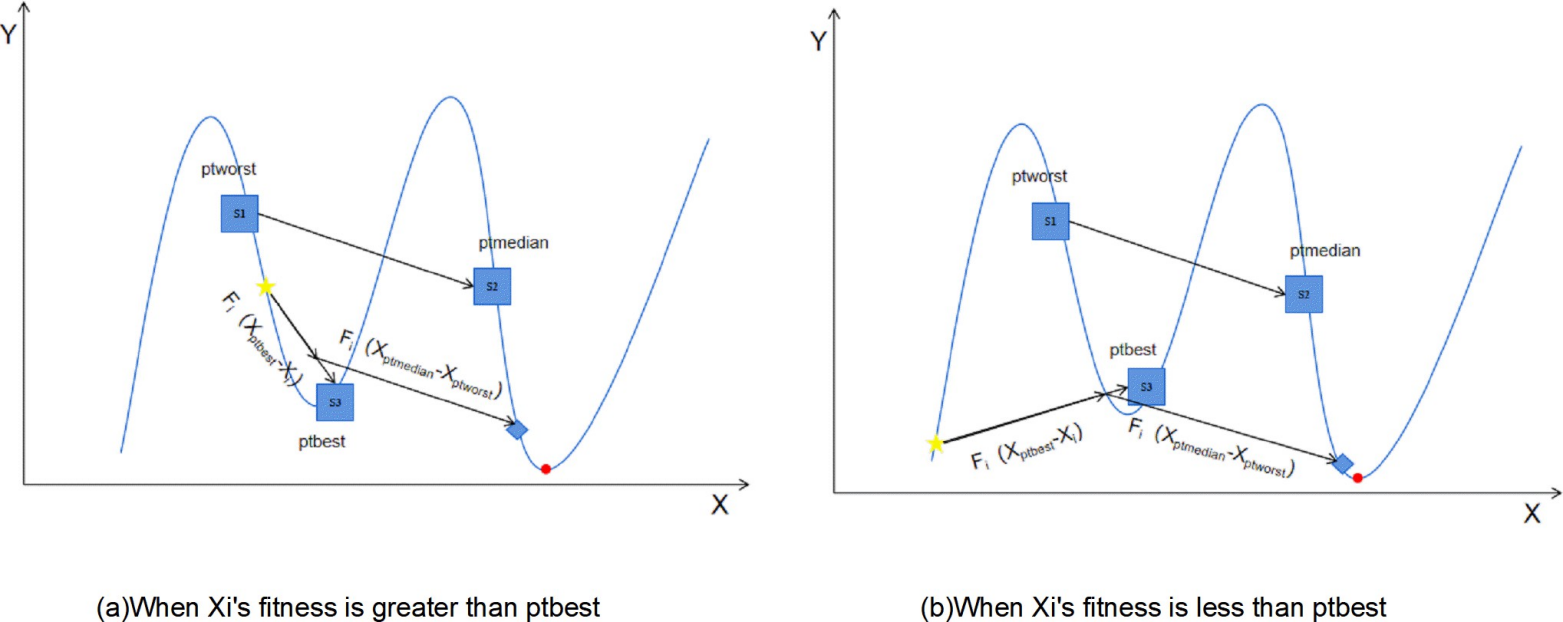
Search strategy for DE/current to ord/1 utilized in this paper.

The search strategy is shown in Fig 1, with stars representing the current individual, circles representing the actual optimal solution,while other three individuals are shown in squares and labeled as S1 to S3. Sort S1, S2, and S3 as ptworst, ptmedian, and ptbest, respectively. L1 and L2 representing the search directions from the current individual to S3 and S1 to S2, respectively.

1. DE/current-to-pbest/1 with external archive

This mutation strategy is used in the JADE algorithm. The DE/current-to-pbest/1 with external archive algorithm possesses excellent global search capability and is able to find relatively optimal solutions in the parameter space. It generates new solution vectors by utilizing the differences between the current solution and the historical solutions, aiding in the exploration of the entire search space.


$$V_{i,G+1}=X_{i,G}+F_{i}\times (X_{pbest,G}-X_{i,G})+F_{i}\times (X_{r1,G}-{\tilde{X}}_{r2,G})$$ (8)

Where, *X*_*i*,*G*_ represents the individual i in the population at generation *G*. *X*_*i*,*pbest*_ refers to the top *p*% elite individuals in the population at generation *G*. The values *r1* is chosen from the individuals in the subpopulation *pop*_*i*_, and the values *r2* is chosen from the individuals in the subpopulation *pop*_*i*_ and the external archive ***A***, where *r1* ≠ *r2* ≠ *pbest*. *pop*_*i*_ indicates the subpopulation.

The DE algorithm’s level of success heavily relies on the choice of the scaling factor *F* and crossover rate *Cr*. These factors hold significant importance as they strongly impact the algorithm’s effectiveness, efficiency, and robustness. Moreover, determining the optimal values for these control parameters across various problems with differing characteristics at various stages of exploitation can be challenging. Hence, the current population impacts the future best control parameters. Therefore, in order to achieve good performance, the proposed algorithm includes new mutation and parameter adaptation methods used in JADE.

For each individual *X*_*i*_ in every generation *G*, the crossover probability *Cr*_*i*_ is generated independently using a normal distribution with an average value of *μCr* and a standard deviation of 0.1.


$${Cr}_{i}={randn}_{i}(\mu Cr,0.1)$$ (9)

Additionally, the crossover probabilities *Cr*_*i*_ in generation g are truncated to fall within the range of [0, 1]. The set *SCr* represents all the successful crossover probabilities. The average *μCr* is initially set to 0.5 and is subsequently updated at the end of each generation to

$$\mu Cr=(1-c)\times \mu Cr+c\times {mean}_{A}(S_{Cr})$$ (10)

where *c* is a positive constant between 0 and 1 and meanA(·) is the usual arithmetic mean.

Similarly, in every generation G, the mutation factor of each individual *X*_*i*_ is independently generated from a Cauchy distribution with a location parameter of *μF* and a scale parameter of 0.1.

$$F_{i}={randc}_{i}(\mu F,0.1)$$ (11)

after that, the generated mutation factors are truncated to 1 if they exceed *F*_*i*_≥1 or regenerated if *F*_*i*_≤0. Take *SF* as the set of all successful mutation factors in the *G*-generation. The location parameter *μF* of the Cauchy distribution is initially set to 0.5 and is subsequently updated at the end of each generation.

$$\mu F=(1-c)\times \mu F+c\times {mean}_{L}(S_{F})$$ (12)

where meanL(·) is the Lehmer mean

$${mean}_{L}(S_{F})=\frac{\sum_{F\in S_{F}}F^{2}}{\sum_{F\in S_{F}}F}$$ (13)

### Multi-population strategy

Multi-population strategy is considered as an effective technique to promote exploration without reducing population diversity. Taking this into consideration, this paper adopts a multiple populations strategy. The detailed process is as follows:

Firstly, this paper divides the entire population into two indicator subpopulations and one reward subpopulation. They are randomly allocated during each generation, with the two indicator subpopulations labeled as *pop*_*1*_ and *pop*_*2*_, and the reward subpopulation labeled as *pop*_*3*_. The indicator subpopulations have the same size, but they are much smaller compared to the reward subpopulation. Let *pop* represent the total population, as shown below.


$$pop=\underset{i=1,2,3}{⋃}{pop}_{i}$$ (14)

Where, *NP* represents the size of pop, while *NP*_*i*_ represents the size of pop_i_. *δ*_*i*_ signifies the ratio between pop_i_ and pop. Therefore, it is expressed as

$${NP}_{i}=\delta_{i}\times NP$$ (15)

$$\sum_{i=1,2,3}\delta_{i}=1$$ (16)

In this paper just let *δ*_1_ = *δ*_2_, firstly, two subpopulations based on different performance metrics are randomly assigned two different DE strategies. The rewarding subpopulation is also randomly assigned to one of the DE strategies. This allocation process is repeated in every generation. As the algorithm proceeds, after every recent generations (*ng)*, this paper determines the most effective DE search strategy (*best*) in the previous iteration based on the ratio between cumulative fitness improvement and cost function evaluation.


$$best={max}_{i=1,2}(\frac{{ns}_{i}}{{\Delta fes}_{i}})$$ (17)

Where, *ns*_*i*_ is the successful numbers of the offspring vectors generated improvement from the most recent *ng* generations of the *i*th composing DE strategy and Δ*fes*_*i*_ is the quantity of function evaluations consumed.

In the following generations (*ng*), subpopulations that demonstrate the best performance in the DE search strategy will be rewarded. The periodic determination of the best DE search strategy and the allocation operator for rewarding subpopulations occurs, with *ng* indicating the interval. This approach guarantees that the optimal mutation strategy utilizes the maximum computational resources available, as intended in this paper.

### Elites regeneration

According to the proximate optimality principle (POP) [43], this paper posits that proficient solutions possess analogous structures that have been employed across nearly all heuristic algorithms.

The sampling method used in elites regeneration primarily stems from EDA, as explicitly employing probabilistic models in EDA offers significant advantages over other types of EAs. Therefore, this paper strives to leverage the benefits of sampling solutions to maintain a high level of population diversity. As a result, the adoption of elites regeneration includes the process of sampling alternative individuals near elite solutions using a probabilistic model following the selection operation.

In [17] suggests that due to the finite variance of the Gaussian distribution. Generating candidate solutions solely through a Gaussian distribution restricts the diversity of the population. Unlike the Gaussian distribution, the Cauchy distribution has a shape that approaches the axes very slowly, to the extent that the expectation does not converge. The results indicate that the Cauchy distribution has an infinite variance, resulting in stronger diversity among the generated individuals. Utilizing Gaussian or Cauchy probability models regenerates new individuals around elite individuals.

Within the domain of probability theory, the frequently utilized continuous probability distribution is the Gaussian distribution, which is scaled by the standard deviation and transformed by the mean value *μ*. The description of the probability density of this distribution is as follows:

$$Gaussian(\mu ,\sigma^{2})=\frac{1}{\sigma }\frac{1}{\sqrt{2\pi }}e^{-\frac{1}{2}{\left(\frac{x-\mu }{\sigma }\right)}^{2}}$$ (18)

The expected value and variance of the Cauchy distribution are indeterminate. This distribution is characterized by two parameters: the location parameter *x*_*0*_, which determines the peak position of the distribution, and the scale parameter, which determines the half-width value at the half-maximum distribution. The equation that describes the probability density function of the Cauchy distribution is as follows:

$$cauchy(x_{0},\gamma )=\frac{1}{\pi }\frac{\gamma }{{\left(x-x_{0}\right)}^{2}+\gamma^{2}}$$ (19)

After performing selection operations, the fitness values of offspring individuals are sorted, and solutions with higher fitness are referred to as high-quality elites.

According to the assumption of the aforementioned population-based optimization process, elite individuals are utilized to re-exploring new individuals. Previous studies have indicated that the likelihood of generating superior solutions in the later stages is lower compared to the early stages. Therefore, the size of the elite population needs to be dynamically adjusted, a direct deterministic linear reduction technique has been introduced. The calculation formula for its size is as follows:

$$EP=round({EP}_{max}-{(EP}_{max}-{EP}_{min})/maxFES\times FES)$$ (20)

Where *EP* represents the scale of the elite population. *EP*_*max*_ is the maximum value for the elite group, specified as *NP* divided by 10. *EP*_*min*_ is the minimum value, set to three individuals. *FES* denotes the number of function evaluations, and *maxFES* represents the maximum function evaluations for the problem dimension. With an increase in the number of *FES*, *EP* gradually decreases from *EP*_*max*_ to *EP*_*min*_. To ensure that the size of the elite population is an integer, the round function is applied to round the value.

Assign the mean parameter *μ* of the Gaussian distribution or the location parameter *x*_*0*_ of the Cauchy distribution to each elite individual. Additionally, it is reasonable to set the standard deviation *σ* of the Gaussian distribution and the scale parameter *γ* of the Cauchy distribution to 0.005 in the 30-D problem through the parameter sensitivity analysis. As a result, during each generation, EP individuals are created using an explicit probabilistic model, such as the Gaussian or Cauchy model. These newly generated individuals are similar to their elite parental solutions, with each individual corresponding to a specific elite parent. The process of creating these individuals is defined as follows:

$$N_{i}=\left\{ \begin{array}{l} Gaussian(E_{i},0.005)\;if\;rand(0,1)<0.5\\ \;Cauchy(E_{i},0.005)\;otherwise \end{array}\right.$$ (21)

Where *E*_*i*_ represents the *i*th elite vector and *N*_*i*_ represents the *i*th newly generated individual.

Next, the newly sampled individuals are compared to their elite parent individuals. If the newly sampled individuals exhibit better fitness values, they replace their parents and become part of the next generation. This mechanism is designed to regenerate individuals near the best solutions found in the search space, thereby exploring promising areas in each generation. The goal is to enhance the convergence speed of the algorithm, guided by the elite individuals, while maintaining population diversity during the sampling process. The pseudo code for EBJADE is shown in Algorithm 1.

In EBJADE, the individual sorting process and elite solution regeneration generate some additional calculations. In order to compare the time complexity of the original DE algorithm and the EBJADE algorithm, it is necessary to evaluate the computational cost. Due to the use of random partitioning in subpopulation partitioning, its computing time is T(NP), its time is complexity O(1). Sort individuals in the population based on fitness, with a time complexity of O(NP×log(NP)). The time complexity when implementing mutation strategies in subpopulations is O(pop_i_×D), and the total complexity for each generation is O(NP×D). The time complexity of regenerating individuals with better ranking in the elite solution regeneration process is O(EP×D). Therefore, the total time complexity of the EBJADE algorithm is O(Gmax×(1+NP×log(NP)+NP×D+EP×D)). Finally simplified as O(Gmax×(NP×D), where Gmax is the maximal generations. The overall time complexity is comparable to the original DE, without increasing the time complexity.

**Algorithm 1** pseudo code of EBJADE

1: Initial parameters including ng, NP, *δ*_*i*_, MaxFes;

2: Initialize the pop randomly distributed in the solution space;

3: Set NP_i_ = *δ*_*i*_×*NP*; μCR = 0.5;μF = 0.5; A = ∅; ns_i_ = 0 and *Δfes*_*i*_ for i = 1, 2, 3;

4: Randomly divide pop into pop_1_,pop_2_ and pop_3_ with respect to their size;

5: Randomly select a subpopulation pop_i_(i = 1, 2, 3) and combine pop_i_ with pop_3_.Let pop_i_ = pop_i_∪pop_3_ and NP_i_ = NP_i_+NP_3_;

6: Set fes = 0;

7: **while** fes ≤ MaxFes do

8: Sort fitness values ${f(X}_{1,G}),{f(X}_{2,G}),\ldots ,{f(X}_{NP,G})$ in ascending order

9: **for** i = 0 to pop_1_ do

10: Generate Cr_i_ = randn_i_(μCr_1_,0.1), F_i_ = randci(μF_1_,0.1)

11: Generate a mutant vector V_i,G_ using Eq (8)

12: Generate a trial vector U_i,G_ using Eq (5)

13: Evaluate trial U_G_; fes++;

14: **end for**

15: **for** i = 0 to pop_2_ do

16: Generate Cr_i_ = randn_i_(μCr_2_,0.1), F_i_ = randci(μF_2_,0.1)

15: Generate a mutant vector V_i,G_ using Eq (7)

16: Generate a trial vector U_i,G_ using Eq (5)

17: Evaluate trial U_G_; fes++;

18: **end for**

19: **if** mod(g,ng) = = 0 **then**

20: $k={max}_{i=1,2}(\frac{{ns}_{i}}{{\Delta fes}_{i}})$;

21: **end if**

22: Randomly partition pop into pop_1_, pop_2_ and pop_3_

23: Let pop_k_ = pop_k_∪pop_3_,(k = 1, 2)

24: Sort fitness values ${f(X}_{1,G}),{f(X}_{2,G}),\ldots ,{f(X}_{NP,G})$ in ascending order

25: Calculate size of the elite population using Eq (20)

26: Select the best EP solutions from the whole population

27: **for** i = 0 to EP do

28: **if** rand ≤ 0.5 **then**

29: Sample a new individual N_i,G_ around the ith

30: Elite individual E_i,G_ from the Gaussian distribution

31: **else**

32: Sample a new individual N_i,G_ around the ith

33: Elite individual E_i,G_ from the Gaussian distribution

34: **end if**

35: Evaluate trial N_G_; fes++;

36: **end for**

37: Randomly remove solutions from A so that |A| ≤ NP

38: Update μCr_1_, μCr_2_ using Eq (10)

39: Update μF_1_, μF_2_ using Eq (12)

40: **end while**

## Numerical experiments and comparisons

In this section, the computational results of the proposed algorithm will be discussed and compared with other state-of-the-art algorithms.

Extensive experiments were conducted using 30 benchmark functions from CEC 2014 to evaluate the effectiveness of our proposed algorithm.These functions can be classified into four groups: unimodal functions (F1-F3), simple multimodal functions (F4-F16), hybrid functions (F17-F22), and composition functions (F23-F30). Detailed descriptions of the functions can be found in [44].

In our proposed algorithm, this paper followed the setting from JADE, where the population size was set to 100, 200, and 400 for 30-D, 50-D, and 100-D, respectively. The recommended parameters for the standard deviation σ of the Gaussian distribution and the scale parameter γ of the Cauchy distribution are used in the ERG framework. These parameter values were chosen because they produced the best optimization results. The experimental results can be divided into three parts to systematically evaluate the optimization performance of the algorithm, while emphasizing the unique components of the proposed algorithm. Firstly, the numerical results and statistical comparisons of the proposed algorithm are described. Furthermore, the impact of parameter sensitivity analysis on algorithm performance was discussed. Lastly, a comparison was made between EBJADE and other state-of-the-art algorithms, including DE variants and variance matrix adaptive evolution strategy (CMA-ES) [45], as well as the winner a hybrid CMA-ES method which combines IPOP-CMA-ES and an iterated local search method (ICMAES-ILS) of CEC2014 [46].

### Experiments setup

To evaluate the algorithm’s performance, this paper utilizes the solution error metric. The obtained best error value and standard deviation less than 10^−8^ are considered as zero [44]. For the CEC2014 benchmark and each function, the maximum number of function evaluations (FEs) is determined as 10000 multiplied by D, where D represents the dimensionality of the problem. This terminal criteria ensures a consistent evaluation across different benchmark functions. To ensure the reliability of the results, each experiment for every function and algorithm is repeated independently 50 times. This repetition helps capture the variation in the algorithm’s performance and provides a more robust evaluation.

To assess the performance of the algorithms [47], two non-parametric statistical hypothesis tests are employed. The first test is the Friedman test, which converts the performance of each algorithm on functions into final rankings. The null hypothesis for this test states that "there is no performance difference among all algorithms," while the alternative hypothesis suggests that "there is a performance difference among all algorithms."

The second test is the Wilcoxon signed-rank test for multiple comparisons, which examines the differences among all algorithms across all functions. The Wilcoxon signed-rank test is employed with a significance level of 0.05 [40]. By utilizing the Wilcoxon signed-rank test, we analyze the variable R^+^. It represents the sum of ranks for functions where the first algorithm outperforms the second algorithm. Similarly, the variable R^−^ represents the sum of ranks for functions where the second algorithm outperforms the first algorithm. A higher rank signifies a greater performance difference. Within each competing function, the values in the "better," "equal," and "worse" categories indicate the number of instances where the first algorithm outperforms, equals, or underperforms the second algorithm, respectively. Based on the test results, a comparative symbol (+, =, and −) is assigned to evaluate the performance of any two algorithms. The plus sign (+) signifies that the proposed algorithm outperforms other algorithms significantly, the equal sign (=) indicates no significant difference between the two algorithms, and the minus sign (−) points that the proposed algorithm significantly underperforms other algorithms. In the context of hypothesis testing, the null hypothesis posits that there is no notable discrepancy in the mean outcomes between the two sample groups. Conversely, the alternative hypothesis proposes that there is a remarkable difference in the mean outcomes among the two samples. The p-value obtained from the test is subsequently compared to a predetermined significance level. If the p-value is less than or equal to the significance level, the null hypothesis is rejected. The p-values corresponding to the significance level are emphasized in bold.

### Numerical results and statistical comparisons of the proposed algorithm

In this section, this paper will discuss the statistical analysis outcomes of applying the Friedman and Multi-Problem Wilcoxon tests to compare the proposed algorithms. The comprehensive statistical results of other algorithms and the proposed algorithms on the CEC2014 benchmark functions can be found in Table 1.

**Table 1:** Average ranks for all algorithms across all problems and all dimensions using CEC2014.

Algorithm	30D	50D	100D	Mean Ranking	Rank
EBJADE	**1.67**	**1.63**	**1.70**	1.67	1
EBJADEwithoutERG	2.73	2.53	2.38	2.55	2
JADE	2.65	2.70	2.52	2.62	3
ord	2.95	3.13	3.40	3.16	4
*Friedman-p-value*	0.000	0.000	0.000		

Table 1 presents the average rankings of the proposed algorithms after conducting the Friedman test using CEC2014, including JADE as the baseline algorithm. The best rankings are highlighted in bold, and the second best rankings are indicated by underlining. From the table, it can be observed that the p-values computed by the Friedman test are less than 0.05 in all dimensions. Based on this, it can be concluded that there are significant differences in the performance of the algorithms.

Besides, it is evident that our proposed algorithm outperforms both the single-strategy algorithm and the algorithm without elite solution regeneration in all dimensions. This demonstrates the effectiveness of multi-strategy adaptation and elite solution regeneration. Our proposed algorithm achieved top ranking in the 10, 50, and 100 dimensions, demonstrating the effectiveness of the dual-strategy mechanism through the use of elite solution regeneration, where it obtained second place in the 50 and 100 dimensions. JADE secured second place in the 30 dimensions, with no significant difference compared to EBJADEwithoutERG. In terms of average ranking, EBJADE took first place, followed by EBJADEwithoutERG and JADE. This observation confirms the positive impact of our proposed mutation strategy on the JADE algorithm.

According to the statistical analysis results in Table 2, this paper can draw the following conclusions: EBJADE outperforms other algorithms using CEC2014, including single-strategy algorithms and algorithms without elite solution regeneration, showing significant advantages in all dimensions. These conclusions were obtained through Wilcoxon tests and statistical analysis conducted between EBJADE and other algorithms. Therefore, based on this test, EBJADE demonstrates clear superiority over single-strategy algorithms and algorithms without elite solution regeneration in all dimensions.

**Table 2:** Wilcoxon’s test results for EBJADE algorithms and other algorithms using CEC2014 functions for D = 30, 50 and 100.

Dimension	Algorithms	R^+^	R^−^	*p-value*	Dec.
30-D	EBJADE vs EBJADEwithoutERG	283	17	**0.000**	+
	EBJADE vs JADE	207	69	**0.036**	+
	EBJADE vs ord	264	61	**0.006**	+
50-D	EBJADE vs EBJADEwithoutERG	289	62	**0.004**	+
	EBJADE vs JADE	316.5	34.5	**0.000**	+
	EBJADE vs ord	287	91	**0.019**	+
100-D	EBJADE vs EBJADEwithoutERG	299	107	**0.029**	+
	EBJADE vs JADE	290	116	**0.048**	+
	EBJADE vs ord	379	27	**0.000**	+

### Parameter analysis

An analysis was conducted to investigate the impact of five adjustable parameters in EBJADE, including the population size *NP*, parameters of DE/current-to-ord/1 mutation strategy *pt*, the ratio *δ*_1_ (as *δ*_1_ = *δ*_2_) between the indicator population and the overall population, the generation gap, *ng*, which is used to periodically determine the mutation strategy based on recent best performance and the scale parameters in elite solution regeneration The performance of EBJADE was evaluated by varying parameter. The analysis aimed to understand how these parameters affect the performance of EBJADE and to find the optimal values for achieving desired results.

In the parameter sensitivity analysis, the candidate values for *δ*_1_ include 0.2, 0.15, 0.25, and 0.3, the candidate values for *ng* include 10, 30, 50, and 80, and the candidate values for Sort vector parameter pt% include 0.05, 0.1, 0.15, 0.2 0.25, 0.3. When analyzing one parameter, the other parameters is set to its default value. The EBJADE with default parameter values is referred to as the standard EBJADE. A Wilcoxon rank−sum test with a significance level of 0.05 was conducted between the standard EBJADE and other EBJADE versions with different parameter values. The symbols "+" "=", and "−" represent better, similar, and worse performance of the standard EBJADE compared to the respective EBJADE versions. The results of the sensitivity analysis for parameters *δ*_1_ and *ng* are recorded in Table 3 and the Sort vector parameter pt% is recorded in Table 4.

**Table 3:** The computation results of EBJADE with different settings of δ_1_ and ng for 30 benchmark functions with 50 experimental runs.

D = 30	EBJADE δ_1_ = 0.15	EBJADE δ_1_ = 0.2	EBJADE δ_1_ = 0.25	EBJADE δ_1_ = 0.3	EBJADE ng = 10	EBJADE ng = 30	EBJADE ng = 50	EBJADE ng = 80	EBJADE STD
F1	9.75e+02	**4.02e+02**	5.06e+02	6.25e+02	7.04e+02	9.41e+02	8.82e+02	7.24e+02	7.66e+02
F2	0.00e+00	0.00e+00	0.00e+00	0.00e+00	1.70e−11	0.00e+00	0.00e+00	0.00e+00	**0.00e+00**
F3	**2.89e−01**	9.34e+00	5.59e+00	9.19e+00	4.51e+00	6.71e+00	4.83e+00	4.69e+00	7.16e−01
F4	9.60e−29	1.27e+00	**6.11e−29**	9.36e−29	3.81e−28	1.84e−28	1.27e+00	1.17e−28	1.55e−28
F5	2.03e+01	2.00e+01	2.00e+01	2.00e+01	2.00e+01	2.00e+01	2.00e+01	2.00e+01	**2.00e+01**
F6	1.02e+01	9.95e+00	1.04e+01	1.03e+01	9.97e+00	1.04e+01	9.92e+00	1.02e+01	**9.58e+00**
F7	0.00e+00	1.48e−04	0.00e+00	0.00e+00	1.53e−09	4.43e−04	5.66e−14	1.34e−09	**0.00e+00**
F8	0.00e+00	0.00e+00	0.00e+00	0.00e+00	0.00e+00	0.00e+00	0.00e+00	0.00e+00	**0.00e+00**
F9	2.36e+01	2.24e+01	2.27e+01	2.21e+01	2.19e+01	2.20e+01	2.26e+01	2.22e+01	**2.15e+01**
F10	**2.50e−03**	6.25e−03	3.75e−03	4.16e−03	8.33e−03	8.33e−03	9.99e−03	8.33e−03	4.58e−03
F11	1.65e+03	**1.51e+03**	1.52e+03	1.53e+03	1.54e+03	1.52e+03	1.54e+03	1.52e+03	1.52e+03
F12	2.68e−01	1.65e−01	1.88e−01	1.74e−01	1.73e−01	1.73e−01	1.68e−01	**1.63e−01**	1.64e−01
F13	2.07e−01	2.10e−01	2.06e−01	2.09e−01	1.96e−01	2.06e−01	2.06e−01	**1.94e−01**	1.99e−01
F14	2.45e−01	2.28e−01	**2.24e−01**	2.26e−01	2.34e−01	2.34e−01	2.33e−01	2.34e−01	2.28e−01
F15	2.99e+00	**2.28e+00**	2.36e+00	2.42e+00	2.31e+00	2.34e+00	2.32e+00	2.35e+00	2.37e+00
F16	9.51e+00	9.53e+00	9.48e+00	9.46e+00	9.37e+00	9.33e+00	9.34e+00	9.40e+00	**9.28e+00**
F17	**1.14e+03**	8.77e+03	1.18e+03	1.23e+03	1.16e+03	4.42e+03	9.44e+03	1.80e+04	1.22e+03
F18	1.10e+02	8.01e+01	7.21e+01	**6.48e+01**	8.37e+01	7.70e+01	8.31e+01	1.61e+02	8.49e+01
F19	4.70e+00	4.80e+00	4.88e+00	**4.58e+00**	4.88e+00	4.66e+00	4.72e+00	4.82e+00	4.80e+00
F20	1.38e+03	1.54e+03	1.35e+03	2.33e+03	1.86e+03	1.73e+03	**1.16e+03**	1.62e+03	1.17e+03
F21	5.23e+02	1.09e+03	9.25e+02	2.06e+03	3.26e+02	7.78e+02	6.45e+03	3.67e+03	**2.90e+02**
F22	1.48e+02	1.15e+02	1.16e+02	1.29e+02	1.20e+02	**1.09e+02**	1.31e+02	1.17e+02	1.27e+02
F23	2.90e+02	2.90e+02	2.90e+02	2.90e+02	2.90e+02	2.90e+02	2.90e+02	2.90e+02	**2.90e+02**
F24	2.01e+02	2.01e+02	2.01e+02	2.01e+02	2.01e+02	2.01e+02	2.01e+02	2.01e+02	**2.01e+02**
F25	2.09e+02	2.09e+02	2.08e+02	2.08e+02	2.09e+02	2.09e+02	2.09e+02	2.09e+02	**2.08e+02**
F26	1.00e+02	1.00e+02	1.00e+02	1.00e+02	1.00e+02	1.00e+02	1.00e+02	1.00e+02	**1.00e+02**
F27	3.75e+02	3.75e+02	3.81e+02	3.83e+02	3.76e+02	3.79e+02	3.81e+02	3.78e+02	**3.73e+02**
F28	4.23e+02	4.23e+02	4.19e+02	**4.19e+02**	4.21e+02	4.26e+02	4.24e+02	4.25e+02	4.22e+02
F29	1.09e+07	1.13e+07	1.06e+07	1.03e+07	9.51e+06	9.93e+06	1.05e+07	**9.46e+06**	1.04e+07
F30	8.03e+02	7.31e+02	7.90e+02	**7.20e+02**	8.25e+02	8.22e+02	8.54e+02	7.63e+02	7.55e+02
+/ = /−	9/21/0	8/21/1	3/26/1	5/22/3	5/24/1	6/23/1	7/23/0	7/22/1	−/−/−

**Table 4:** The computation results of EBJADE with different settings of pt for 30 benchmark functions with 50 experimental runs.

D = 30	EBJADE pt = 0.05	EBJADE pt = 0.1	EBJADE pt = 0.15	EBJADE pt = 0.2	EBJADE pt = 0.25	EBJADE STD
F1	1.91E+03	1.28E+03	**5.06E+02**	1.12E+03	5.63E+02	7.66E+02
F2	1.77E-08	0.00E+00	0.00E+00	1.40E-14	0.00E+00	**0.00E+00**
F3	1.04E+01	1.94E+01	5.59E+00	1.00E+01	8.40E+00	**7.16E-01**
F4	6.12E-28	1.13E-28	**6.11E-29**	1.27E+00	1.06E-28	1.55E-28
F5	2.00E+01	2.00E+01	2.00E+01	2.00E+01	2.00E+01	**2.00E+01**
F6	9.24E+00	**9.01E+00**	1.04E+01	9.29E+00	9.36E+00	9.58E+00
F7	5.45E-04	2.46E-04	0.00E+00	1.16E-10	2.41E-13	**0.00E+00**
F8	0.00E+00	0.00E+00	0.00E+00	0.00E+00	0.00E+00	**0.00E+00**
F9	2.40E+01	2.30E+01	2.27E+01	2.24E+01	2.15E+01	**2.15E+01**
F10	1.08E-02	1.04E-02	**3.75E-03**	1.04E-02	4.16E-03	4.58E-03
F11	**1.51E+03**	1.50E+03	1.52E+03	1.57E+03	1.52E+03	1.52E+03
F12	1.66E-01	1.62E-01	1.88E-01	**1.58E-01**	1.73E-01	1.64E-01
F13	2.09E-01	2.04E-01	2.06E-01	2.07E-01	2.01E-01	**1.99E-01**
F14	2.52E-01	2.45E-01	**2.24E-01**	2.29E-01	2.39E-01	2.28E-01
F15	2.43E+00	2.34E+00	2.36E+00	2.35E+00	**2.34E+00**	2.37E+00
F16	9.31E+00	9.38E+00	9.48E+00	9.31E+00	9.34E+00	**9.28E+00**
F17	1.28E+03	1.36E+03	**1.18E+03**	1.29E+03	3.39E+04	1.22E+03
F18	1.20E+02	1.21E+02	**7.21E+01**	9.75E+01	9.32E+01	8.49E+01
F19	4.96E+00	4.95E+00	4.88E+00	4.74E+00	**4.79E+00**	4.80E+00
F20	1.53E+03	1.98E+03	1.35E+03	1.84E+03	**1.08E+03**	1.17E+03
F21	5.83E+03	3.73E+03	9.25E+02	3.16E+03	1.13E+03	**2.90E+02**
F22	1.46E+02	1.07E+02	1.16E+02	**1.06E+02**	1.18E+02	1.27E+02
F23	2.90E+02	2.90E+02	2.90E+02	2.90E+02	2.90E+02	**2.90E+02**
F24	2.01E+02	2.01E+02	2.01E+02	2.01E+02	2.01E+02	**2.01E+02**
F25	2.08E+02	2.09E+02	2.08E+02	2.09E+02	2.09E+02	**2.08E+02**
F26	1.00E+02	1.00E+02	1.00E+02	1.00E+02	1.00E+02	**1.00E+02**
F27	3.74E+02	**3.66E+02**	3.81E+02	3.75E+02	3.81E+02	3.73E+02
F28	4.22E+02	4.20E+02	**4.19E+02**	4.22E+02	4.23E+02	4.22E+02
F29	1.20E+07	1.17E+07	1.06E+07	1.05E+07	1.08E+07	**1.04E+07**
F30	9.16E+02	8.97E+02	7.90E+02	8.18E+02	8.06E+02	**7.55E+02**
+/ = /−	21/7/2	16/10/4	12/11/7	19/6/5	14/12/4	−/−/−

The data provided in Table 3 reveals that EBJADE is not sensitive to parameters *δ*_1_ and *ng* for many benchmark functions, including F1, F8, F13, and F18-F25. Additionally, EBJADE versions with different parameter values rarely outperform the standard EBJADE, indicating that the parameter settings of the standard EBJADE are reasonable. However, it is worth noting that for function F18, EBJADE with *δ*_1_ = 0.3 surpasses the standard EBJADE.

The data in Table 4 indicates that the standard parameter value pt = 0.3 yields better values than other candidate solutions, and EBJADE is sensitive to the parameter pt. In addition, EBJADE versions with different parameter values are rarely superior to standard EBJADE, indicating that the parameter settings of standard EBJADE are reasonable.

In parameter sensitivity analysis, the candidate values for *NP* include 50, 100,150, 200, 300, 400 and the candidate values for scale parameters *σ*^2^, *γ*, include 0.001, 0.005, 0.01, 0.05. Using the Friedman test for ranking, it converts the performance of algorithms with different parameters on the function into the final ranking. The results of the sensitivity analysis for parameters *NP* is recorded in Table 5 and the scale parameters *σ*^2^, *γ* are recorded in Table 6. Tables 5 and 6 show the rank values of all variables, the most competitive result is shown in boldface.

**Table 5:** The computation results of EBJADE with different settings of NP.

Dimension\NP	50	100	150	200	300	400	*Friedman-p-value*
30-D	3.93	**3.02**	3.20	3.33	3.55	3.97	0.163
50-D	4.17	3.45	3.45	**3.20**	3.42	3.32	0.326
100-D	4.58	3.73	3.18	3.32	3.23	**2.95**	0.007

**Table 6:** The computation results of EBJADE with different settings of scale parameters.

Dimension\scale parameters	0.001	0.005	0.01	0.05	*Friedman-p-value*
30-D	2.38	**1.88**	3.05	2.68	0.001
50-D	2.82	2.38	**1.88**	2.92	0.002
100-D	2.57	2.70	2.55	**2.18**	0.409

The number of function variables is a key parameter that determines the difficulty of identifying the global optimum. Therefore, high-dimensional functions are more difficult to solve than low dimensional functions. In different dimensions of problems. To determine the optimal standard deviation and scale parameter values for each dimension, different parameter values are used to evaluate the results of the algorithm for three different dimensions of problems.

The data provided in Table 5 indicates that whether NP is too large or too small greatly affects the results.It is reasonable to set NP to 100, 200, 400 at 30-D, 50-D, and 100-D respectively. Table 6 summarizes the rank values measured at dimensions 30, 50, and 100, using 50 independent runs for each function. The optimal values for the 30-D, 50-D, and 100-D groups are 0.005, 0.01, and 0.05, respectively. These results indicate that increasing dimensionality requires a relatively large sampling range to achieve optimal performance.

### Comparison against state-of-the-art DE variants

All parameter settings of these DE variants are listed in Table 7. Table 8 presents the average rankings of the compared algorithms after conducting the Friedman test on dimensions D = 30, 50, and 100 using CEC2014. It is evident from the table that the calculated p-values from the Friedman test are less than 0.05 for all dimensions. Therefore, this paper can conclude that there are significant differences in performance among the algorithms.

**Table 7:** Parameter settings.

Algorithms	Parameters initial settings
EBJADE	NP = 100, 200,400, μCR = μF = 0.5, c = 0.1, pt = 0.3, δ_i_ = 0.1, γ = σ^2^ = 0.005,0.01,0.05
CoDE	NP = 30, [F = 1.0, CR = 0.1], [F = 1.0, CR = 0.9], [F = 0.8, CR = 0.2]
jDE	NP = 100; τ1 = τ2 = 0.1; Fl = 0.1; Fu = 0.9
SaJADE	NP = 100, 200, 400, μF = μCR = μ_s_ = 0.5; c = 0.1; p = 0.05
EPSDE	NP = 50; F ∈ [0.4, 0.9] and CR ∈ [0.1, 0.9] with stepsize = 0.1
SHADE	NP = 100, μCR = μF = 0.5, H = 100, c = 0.1, p = 0.2
L-SHADE	NP = 18×D, μCR = μF = 0.5, H = 100, c = 0.1, p = 0.2

It is interesting to note that EBJADE ranks third in 30 dimensions, but achieves the second only to L-SHADE in other dimensions. Although there is a slight difference in average rankings between EBJADE (ranked second) and the first-ranked L-SHADE, it is not significant. This indicates that EBJADE consistently performs well across multiple dimensions. Compared to excellent DE variants, it has certain competitiveness.

**Table 8:** Average ranks for all algorithms across all problems for D = 30, 50, and 100 using CEC2014.

Algorithm	30D	50D	100D	Mean Ranking	Rank
L-SHADE	**2.40**	**2.68**	**2.40**	2.49	1
EBJADE	3.57	2.83	2.57	2.99	2
SHADE	3.20	2.98	3.18	3.12	3
SaJADE	4.13	3.45	3.53	3.70	4
jDE	4.87	5.27	4.50	4.88	5
EPSDE	3.98	5.13	5.32	4.81	6
coDE	5.17	5.65	6.50	5.77	7
*Friedman-p-value*	0.000	0.000	0.000		

According to the Wilcoxon test shown in Table 9, EBJADE outperforms EPSDE significantly in all dimensions. In the 30-dimensional case, only EPSDE and EBJADE exhibit a significant difference. As the dimension increases, jDE and CoDE show significant differences from EBJADE in the 50 and 100 dimensions. In the 100-dimensional case, SaJADE also demonstrates a significant difference. However, SHADE and L-SHADE does not exhibit any significant differences across all dimensions.

**Table 9:** Wilcoxon’s test results for EBJADE algorithms and other state-of-the-art DE variants using CEC2014 functions.

Dimension	Algorithms	R^+^	R^−^	*p-value*	Dec.
30-D	EBJADE vs jDE	212	166	0.581	=
	EBJADE vs SaJADE	227	98	0.083	=
	EBJADE vs coDE	250.5	127.5	0.140	=
	EBJADE vs EPSDE	254	97	**0.046**	+
	EBJADE vs SHADE	83.5	192.5	0.097	=
	EBJADE vs L-SHADE	99	252	0.052	=
50-D	EBJADE vs jDE	289	62	**0.004**	+
	EBJADE vs SaJADE	254	124	0.118	=
	EBJADE vs coDE	361	45	**0.000**	+
	EBJADE vs EPSDE	328	50	**0.001**	+
	EBJADE vs SHADE	247	104	0.069	=
	EBJADE vs L-SHADE	132.5	218.5	0.275	=
100-D	EBJADE vs jDE	353	82	**0.002**	+
	EBJADE vs SaJADE	298	108	**0.031**	+
	EBJADE vs coDE	413	22	**0.000**	+
	EBJADE vs EPSDE	378	28	**0.000**	+
	EBJADE vs SHADE	275	121	0.062	=
	EBJADE vs L-SHADE	139	267	0.154	=

S10–S12 Tables present the mean and standard error results of advanced DE algorithm variants on 30-D, 50-D, and 100-D CEC 2014 benchmark functions, respectively. The results indicate that the EBJADE algorithm improves exploratory capabilities and enhances the performance of the algorithm. It either significantly outperforms other advanced algorithms or performs comparably. In the 30-D tests, more than half of the functions exhibit better performance compared to jDE, CoDE, and EPSDE algorithms. While five to six individual functions perform worse, our proposed algorithm outperforms or is comparable to SaJADE and SHADE algorithms for two-thirds of the functions. Compared to L-SHADE, one-third of the functions are better, with comparable performance. Notably, it surpasses all other advanced algorithms for the unimodal function F2 and the multimodal functions F11, F12, F15, and F16.

In the 50-D and 100-D tests, the effectiveness of the EBJADE algorithm is further enhanced. In the 50-D test, it outperforms jDE, CoDE, and EPSDE algorithms in more than 20 functions. Specifically, it performs better in 24 functions compared to CoDE and 23 functions compared to EPSDE algorithms. While the improvement over the SaJADE algorithm is not as significant, it still performs better than in the 30-D test. It shows an increase of 6 functions compared to the SHADE algorithm. Compared with L-SHADE, the proposed algorithm has improved performance. In the 100-D test, jDE, CoDE, and EPSDE algorithms show improvement in 2, 3, and 3 functions respectively, while SaJADE improves in one function and SHADE improves in two functions. Performance comparable to 50D Compared with L-SHADE. The results demonstrate that the EBJADE algorithm performs better than other algorithms in many multimodal functions, and its performance becomes more significant as the dimension increases.

**Fig 2:**
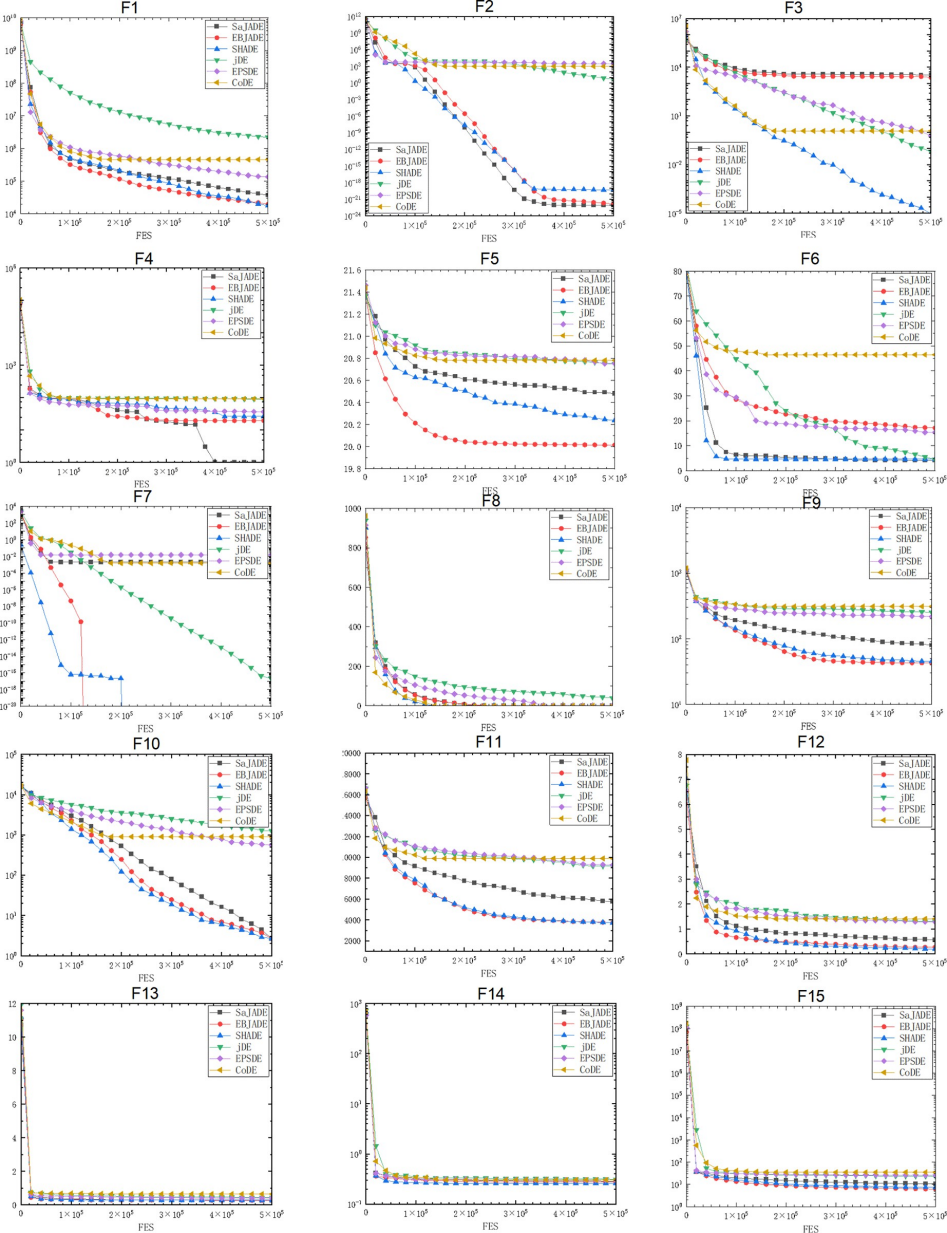
Convergence graphs of CEC2014 F1-F15 benchmark functions with 50 variable.

**Fig 3:**
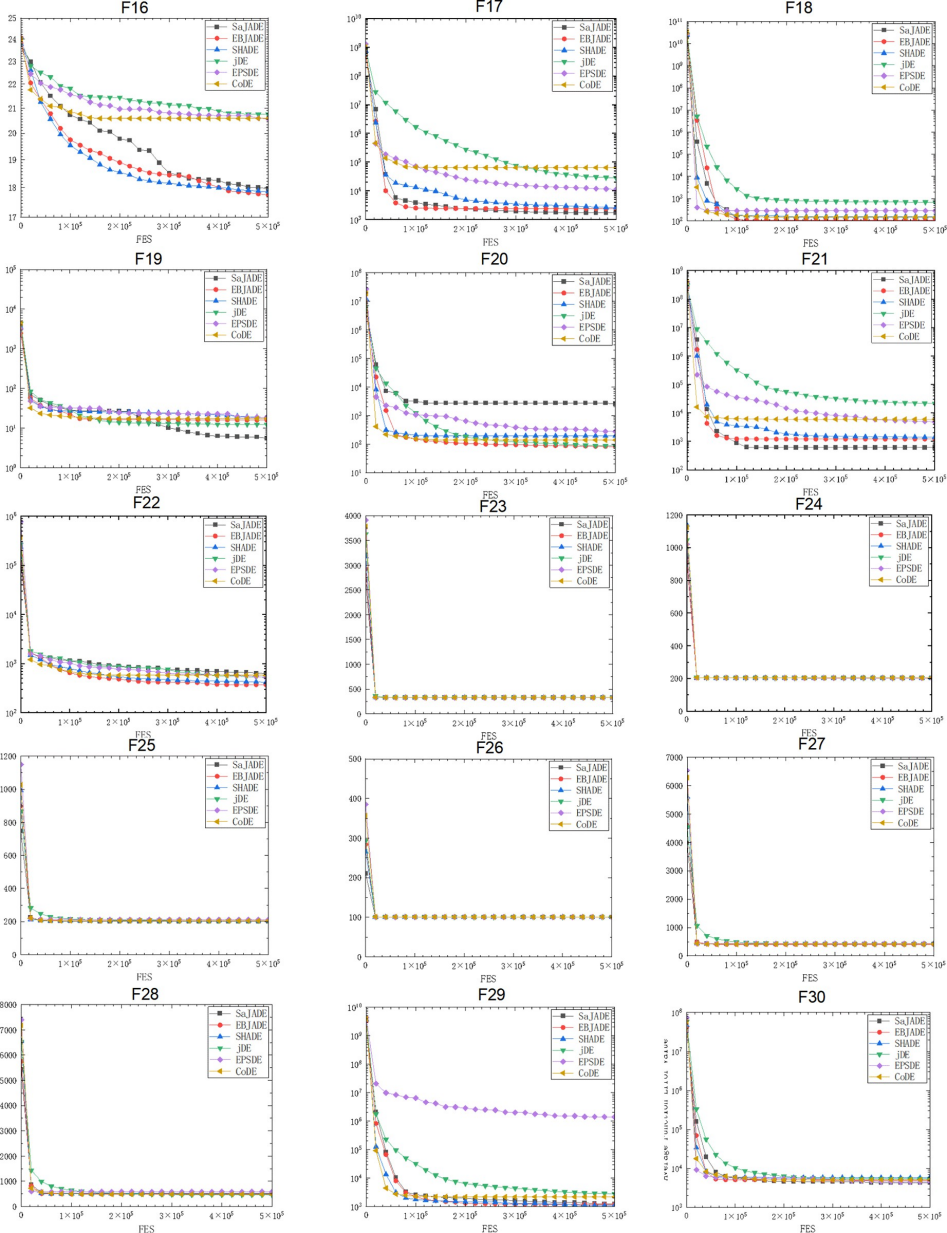
Convergence graphs of CEC2014 F16-F30 benchmark functions with 50 variable.

In Figs 2 and 3, EBJADE has the fastest convergence speed on 11 out of 30 functions. Its convergence speed is higher than all other algorithms on functions F1, F5, F7, F9, f15, and F17. The convergence speed of EBJADE and SHADE on functions F9, F10, F11, F12, F15, F16, and F22 is very close, ranking first, and slightly faster than other algorithms. The convergence rates of EBJADE and saJADE on functions F2, F8, and F18 are not significantly different, and they are also superior to other algorithms. The convergence speed of EBJADE ranks second among the two functions. On functions F4 and F24, only saJADE converges slightly faster than EBJADE. EBJADE performs slightly worse on functions F3 and F6, while all algorithms have similar convergence rates on functions F13, F14, F19, F23-F28, and F30. The algorithm performance of SHADE and saJADE is relatively good, with SHADE in the first or parallel first position among 10 functions, and saJADE in the first or parallel first position among 8 functions.

### Comparison against CMA-ES and variants

In this section, we compare the EBJADE with CMA-ES and its variant combines IPOP-CMA-ES and an iterated local search method (ICMAES-ILS) using CEC2014 for dimensions D = 50 and 100. For ICMAES-ILS, the initial step size is set to 120. The Wilcoxon’s Test results for EBJADE algorithms and other state-of-the-art CEA-ES algorithms in Table 10.

**Table 10:** Wilcoxon’s test results for EBJADE algorithms and other state-of-the-art CEA-ES algorithms using CEC2014 functions.

Dimension	Algorithms	R^+^	R^−^	*p-value*	Dec.
50-D	EBJADE vs CMAES	312	153	0.102	=
	EBJADE vs ICMAESILS	138	213	0.341	=
100-D	EBJADE vs CMAES	263	202	0.530	=
	EBJADE vs ICMAESILS	187	238	0.510	=

S13 and S14 Tables present the mean and standard error results of CMA-ES and its variant on 50-D, and 100-D CEC 2014 benchmark functions, respectively. Its performance is somewhat competitive with the comparative algorithm performance. In the 50-D tests, nearly half of the functions showed better or close performance than the CMA-ES and ICMAES-ILS algorithms. Two-thirds of the proposed algorithms have better results compared to CMAES, and one-third of the proposed algorithms have better results compared to ICMAES-ILS. In the 50-D tests, it also has a similar competitiveness. Notably, it surpasses all other advanced algorithms for the function F8 the functions F10, F16, F18,F21, and F28.

As can be seen from the comparison in Table 10, all statistics are not significant, which means that EBJADE does not significantly outperform the comparison algorithms.A larger R^+^ compared with R^−^ indicates that one algorithm performs better than another algorithm in terms of the number of functions it performs worse than the other algorithm.CMAES has a smaller R^+^ in all dimensions, while ICMAES-ILS has a larger R^+^, indicating that EBJADE’s performance on CEC2014 is slightly better than CMAES but slightly inferior to ICMAES-ILS, and more pronounced on 50D. However, overall, its optimization performance on the CEC 2014 benchmark is similar. The numerical routine of covariance matrix decomposition usually requires O(n^3^) calculation in an n-dimensional search space [48]. S15 Table presents the computating time comparison comparing CMAES and EBJADE on 30 benchmark functions of 50-D and 100-D in CEC2014, respectively. Comparare in Windows 10 on Intel (R) Core (TM) i7-6700 2.60 GHz processor and 16gb RAM, using c + + programming language and Code Block writing algorithm. Therefore, from the perspective of time complexity and computation time, the EBJADE algorithm has certain competitiveness.

## Conclusion

To improve the overall performance of the basic DE algorithm, introducing a new mutation strategy to improve the JADE algorithm, introducing multiple populations mechanisms to allocate computing resources, and a regeneration mechanism for elite solutions. The combination of JADE’s strategy and new strategies, one with strong exploration ability and the other with strong exploitation ability. The proposed mutation strategy enhances global and local search capabilities and improves convergence speed. According to the POP, exploring the neighborhood of exceptional individuals represents a highly promising search space capable of generating superior solutions.

Numerous experiments on the CEC 2014 benchmark suite have shown that EBJADE outperforms several other efficient and popular DE variants, as well as CMA-ES and variants, with no significant differences. Experimental analysis shows that different mutation strategies generally require different control parameters. Two new parameters have been introduced in EBJADE, namely the ratio of the indicator population to the overall population, and the generation gap used to periodically determine the nearest best mutation strategy. Experiments have shown that on most benchmark functions, EBJADE is not sensitive to these two new parameters.

## Supporting information

S1 Table Comparison of the effectiveness of the mechanisms using CEC2014 functions with 30 variables. (PDF)

S2 Table Comparison of the effectiveness of the mechanisms using CEC2014 functions with 50 variables. (PDF)

S3 Table Comparison of the effectiveness of the mechanisms using CEC2014 functions with 100 variables. (PDF)

S4 Table The computation results of EBJADE with different settings of scale parameters for CEC2014 functions with 30 variables. (PDF)

S5 Table The computation results of EBJADE with different settings of scale parameters for CEC2014 functions with 50 variables. (PDF)

S6 Table The computation results of EBJADE with different settings of scale parameters for CEC2014 functions with 100 variables. (PDF)

S7 Table The computation results of EBJADE with different settings of NP for CEC2014 functions with 30 variables. (PDF)

S8 Table The computation results of EBJADE with different settings of NP for CEC2014 functions with 50 variables. (PDF)

S9 Table The computation results of EBJADE with different settings of NP forCEC2014 functions with 100 variables. (PDF)

S10 Table Test results for EBJADE algorithms and other state-of-the-art DE variants using CEC2014 functions with 30 variables. (PDF)

S11 Table Test results for EBJADE algorithms and other state-of-the-art DE variants using CEC2014 functions with 50 variables. (PDF)

S12 Table Test results for EBJADE algorithms and other state-of-the-art DE variants using CEC2014 functions with 100 variables. (PDF)

S13 Table Test results for EBJADE algorithms and other state-of-the-art CEA-ES algorithms using CEC2014 functions with 50 variables. (PDF)

S14 Table Test results for EBJADE algorithms and other state-of-the-art CEA-ES algorithms using CEC2014 functions with 100 variables. (PDF)

S15 Table Computing times results for EBJADE algorithms and CEA-ES algorithms using CEC2014 functions with 50 and 100 variables. (PDF)
