# Supplementary material for: A novel differential evolution algorithm with multi-population and elites regeneration
Source: PLoS One. 2024 Apr 25;19(4):e0302207. doi: 10.1371/journal.pone.0302207 (PMC11045134; doi:10.1371/journal.pone.0302207)
Supplement: S1 Table — (PDF) [file pone.0302207.s001.pdf]

D=30	**ord**	**JADE**	**EBJADE withoutERG**	**EBJADE**
Fi	**Mean(St.D)**	**Mean(St.D)**	**Mean(St.D)**	**Mean(St.D)**
F1	3.65e+03(3.91e+03)	4.87e+02(1.10e+03)	1.46e+03(1.86e+03)	7.66e+02(1.09e+03)
F2	6.14e-25(2.44e-24)	0.00e+00(0.00e+00)	0.00e+00(0.00e+00)	0.00e+00(0.00e+00)
F3	1.34e+01(1.17e+01)	8.45e-01(9.82e-01)	3.72e-01(6.51e-01)	7.16e-01(1.26e+00)
F4	6.72e-01(3.72e-01)	1.62e+01(3.58e+01)	7.97e-02(5.58e-01)	1.55e-28(1.67e-28)
F5	2.03e+01(3.26e-02)	2.03e+01(2.94e-02)	2.03e+01(3.32e-02)	2.00e+01(1.71e-04)
F6	1.16e+01(1.04e+00)	8.93e+00(2.73e+00)	9.65e+00(2.48e+00)	9.58e+00(2.19e+00)
F7	3.67e-09(2.56e-08)	1.97e-04(1.38e-03)	1.86e-08(1.30e-07)	0.00e+00(0.00e+00)
F8	0.00e+00(0.00e+00)	0.00e+00(0.00e+00)	0.00e+00(0.00e+00)	0.00e+00(0.00e+00)
F9	2.70e+01(4.28e+00)	2.67e+01(4.54e+00)	2.32e+01(3.79e+00)	2.15e+01(4.38e+00)
F10	1.25e-03(4.94e-03)	9.16e-03(1.33e-02)	4.58e-03(9.58e-03)	4.58e-03(8.62e-03)
F11	1.67e+03(2.29e+02)	1.63e+03(2.27e+02)	1.69e+03(2.21e+02)	1.52e+03(2.08e+02)
F12	2.74e-01(3.83e-02)	2.67e-01(3.89e-02)	2.67e-01(3.88e-02)	1.64e-01(3.01e-02)
F13	2.04e-01(2.77e-02)	2.30e-01(4.03e-02)	2.17e-01(3.98e-02)	1.99e-01(3.10e-02)
F14	2.41e-01(3.08e-02)	2.41e-01(3.32e-02)	2.48e-01(2.87e-02)	2.28e-01(4.04e-02)
F15	3.18e+00(3.26e-01)	3.17e+00(3.65e-01)	2.93e+00(4.34e-01)	2.37e+00(3.71e-01)
F16	9.37e+00(3.59e-01)	9.31e+00(3.95e-01)	9.46e+00(4.08e-01)	9.28e+00(4.62e-01)
F17	1.06e+03(3.97e+02)	1.30e+03(3.33e+02)	1.30e+03(4.31e+02)	1.22e+03(4.22e+02)
F18	1.67e+03(3.62e+03)	1.94e+02(7.67e+02)	8.57e+01(3.37e+01)	8.49e+01(3.02e+01)
F19	4.97e+00(6.58e-01)	4.36e+00(7.71e-01)	4.84e+00(6.72e-01)	4.80e+00(7.44e-01)
F20	3.51e+03(2.38e+03)	3.21e+03(2.03e+03)	1.63e+03(2.95e+03)	1.17e+03(2.66e+03)
F21	1.46e+04(4.70e+04)	1.65e+04(4.16e+04)	3.25e+02(1.14e+02)	2.90e+02(2.90e+02)
F22	1.54e+02(5.73e+01)	1.45e+02(7.28e+01)	1.36e+02(6.64e+01)	1.27e+02(6.51e+01)
F23	2.90e+02(1.93e-13)	2.90e+02(1.78e-13)	2.90e+02(1.89e-13)	2.90e+02(1.89e-13)
F24	2.01e+02(5.75e-02)	2.01e+02(1.28e-01)	2.01e+02(7.43e-02)	2.01e+02(1.20e-01)
F25	2.09e+02(2.88e+00)	2.08e+02(1.68e+00)	2.09e+02(2.39e+00)	2.08e+02(1.48e+00)
F26	1.00e+02(3.18e-02)	1.00e+02(3.78e-02)	1.00e+02(3.55e-02)	1.00e+02(3.96e-02)
F27	3.87e+02(3.42e+01)	3.95e+02(2.14e+01)	3.74e+02(4.58e+01)	3.73e+02(4.19e+01)
F28	4.18e+02(5.78e+00)	4.21e+02(8.38e+00)	4.24e+02(8.96e+00)	4.22e+02(8.57e+00)
F29	1.02e+07(5.12e+06)	1.04e+07(5.64e+06)	1.19e+07(3.54e+06)	1.04e+07(4.95e+06)
F30	7.60e+02(1.86e+02)	7.07e+02(2.10e+02)	8.43e+02(2.60e+02)	7.55e+02(2.21e+02)
rank	4	2	3	1
