# Supplementary material for: A novel differential evolution algorithm with multi-population and elites regeneration
Source: PLoS One. 2024 Apr 25;19(4):e0302207. doi: 10.1371/journal.pone.0302207 (PMC11045134; doi:10.1371/journal.pone.0302207)
Supplement: S2 Table — (PDF) [file pone.0302207.s002.pdf]

D=50	**ord**	**JADE**	**EBJADE withoutERG**	**EBJADE**
Fi	**Mean(St.D)**	**Mean(St.D)**	**Mean(St.D)**	**Mean(St.D)**
F1	9.87e+04(4.18e+04)	2.57e+04(1.47e+04)	3.48e+04(2.26e+04)	2.13e+04(1.12e+04)
F2	3.26e+03(2.63e+03)	1.40e-22(1.58e-22)	3.55e-22(2.47e-22)	2.43e-22(3.04e-22)
F3	4.26e+03(1.81e+03)	4.40e+03(1.84e+03)	2.04e+03(2.27e+03)	2.50e+03(2.35e+03)
F4	9.43e+01(7.45e+00)	1.62e+01(3.58e+01)	2.96e+01(4.13e+01)	1.37e+01(3.40e+01)
F5	2.05e+01(2.89e-02)	2.05e+01(2.91e-02)	2.05e+01(2.77e-02)	2.00e+01(1.72e-03)
F6	2.67e+01(2.20e+00)	2.24e+01(4.50e+00)	2.31e+01(4.91e+00)	2.17e+01(7.39e+00)
F7	1.31e-03(3.20e-03)	3.45e-04(1.71e-03)	5.42e-04(2.17e-03)	0.00e+00(0.00e+00)
F8	3.08e-08(1.00e-08)	1.11e-11(5.07e-12)	4.64e-12(6.90e-12)	1.46e-11(1.87e-11)
F9	8.61e+01(8.42e+00)	8.23e+01(7.81e+00)	5.95e+01(9.66e+00)	4.52e+01(7.43e+00)
F10	8.84e+00(1.20e+00)	4.35e+00(6.88e-01)	7.18e+00(1.32e+00)	2.47e+00(8.64e-01)
F11	5.53e+03(2.69e+02)	5.58e+03(3.57e+02)	5.03e+03(2.93e+02)	3.94e+03(3.44e+02)
F12	5.31e-01(5.21e-02)	5.33e-01(5.13e-02)	5.15e-01(4.82e-02)	2.96e-01(4.31e-02)
F13	2.95e-01(3.41e-02)	2.90e-01(3.20e-02)	2.90e-01(3.09e-02)	2.88e-01(3.69e-02)
F14	3.01e-01(2.31e-02)	2.86e-01(2.40e-02)	2.92e-01(2.48e-02)	2.82e-01(2.92e-02)
F15	1.01e+01(8.20e-01)	9.93e+00(5.95e-01)	8.30e+00(8.76e-01)	6.05e+00(6.54e-01)
F16	1.84e+01(4.32e-01)	1.84e+01(4.31e-01)	1.83e+01(8.76e-01)	1.80e+01(4.10e-01)
F17	2.13e+03(6.85e+02)	2.12e+03(4.52e+02)	2.25e+03(5.31e+02)	2.17e+03(5.24e+02)
F18	1.17e+02(2.12e+01)	1.37e+02(3.03e+01)	1.40e+02(3.87e+01)	1.32e+02(2.58e+01)
F19	1.77e+01(1.03e+01)	1.37e+01(9.01e+00)	1.57e+01(9.00e+00)	1.62e+01(9.34e+00)
F20	9.23e+03(7.09e+03)	8.47e+03(6.54e+03)	2.71e+03(5.81e+03)	1.89e+03(4.84e+03)
F21	9.14e+02(3.46e+02)	1.11e+03(3.00e+02)	1.06e+03(3.19e+02)	1.00e+03(3.06e+02)
F22	6.12e+02(1.52e+02)	5.92e+02(1.43e+02)	5.63e+02(1.57e+02)	3.58e+02(1.15e+02)
F23	3.27e+02(2.96e-13)	3.27e+02(3.41e-13)	3.27e+02(3.41e-13)	3.27e+02(3.26e-13)
F24	2.03e+02(1.65e-01)	2.03e+02(1.78e-01)	2.03e+02(2.09e-01)	2.03e+02(1.95e-01)
F25	2.03e+02(3.27e+00)	2.05e+02(2.46e+00)	2.04e+02(2.79e+00)	2.05e+02(1.79e+00)
F26	1.00e+02(3.13e-02)	1.00e+02(3.29e-02)	1.00e+02(3.23e-02)	1.00e+02(3.51e-02)
F27	4.01e+02(3.79e+00)	4.16e+02(9.58e+00)	4.13e+02(1.02e+01)	4.10e+02(9.76e+00)
F28	5.11e+02(1.54e+01)	5.00e+02(1.41e+01)	4.99e+02(1.77e+01)	4.71e+02(1.60e+01)
F29	1.01e+03(1.73e+02)	9.79e+02(2.80e+01)	9.95e+02(1.58e+02)	9.78e+02(3.96e+01)
F30	4.34e+03(1.15e+03)	4.97e+03(1.02e+03)	4.50e+03(9.17e+02)	4.38e+03(1.11e+03)
rank	4	3	2	1
