# Supplementary material for: A novel differential evolution algorithm with multi-population and elites regeneration
Source: PLoS One. 2024 Apr 25;19(4):e0302207. doi: 10.1371/journal.pone.0302207 (PMC11045134; doi:10.1371/journal.pone.0302207)
Supplement: S3 Table — (PDF) [file pone.0302207.s003.pdf]

D=100	**ord**	**JADE**	**EBJADE withoutERG**	**EBJADE**
Fi	**Mean(St.D)**	**Mean(St.D)**	**Mean(St.D)**	**Mean(St.D)**
F1	2.90e+06(7.28e+05)	2.16e+05(4.75e+04)-	2.50e+05(6.44e+04)	2.26e+05(7.04e+04)
F2	1.21e+04(5.21e+03)	3.63e-21(1.26e-21)-	1.46e-20(1.63e-20)	1.13e-18(6.08e-18)
F3	8.77e+03(3.98e+03)	8.71e+03(2.07e+03)+	4.28e+03(4.76e+03)	4.54e+03(4.77e+03)
F4	2.76e+02(4.08e+01)	1.14e+02(4.53e+01)+	9.54e+01(5.20e+01)	8.32e+01(3.47e+01)
F5	2.08e+01(2.20e-02)	2.08e+01(1.81e-02)+	2.08e+01(2.57e-02)	2.06e+01(2.80e-02)
F6	7.81e+01(6.12e+00)	4.56e+01(2.87e+01)-	6.55e+01(2.68e+01)	7.33e+01(1.75e+01)
F7	2.66e-03(8.04e-03)	3.94e-04(1.93e-03)+	7.38e-04(3.62e-03)	3.55e-17(6.45e-17)
F8	1.54e+01(1.25e+00)	7.81e+00(7.20e-01)+	1.06e+01(1.26e+00)	7.13e+00(1.37e+00)
F9	2.78e+02(1.69e+01)	2.62e+02(1.71e+01)+	2.27e+02(2.01e+01)	1.14e+02(1.38e+01)
F10	9.16e+02(7.90e+01)	7.37e+02(6.72e+01)+	8.36e+02(6.61e+01)	2.51e+02(8.11e+01)
F11	1.75e+04(3.67e+02)	1.76e+04(3.64e+02)+	1.64e+04(4.15e+02)	1.16e+04(6.30e+02)
F12	1.04e+00(5.31e-02)	1.01e+00(5.91e-02)+	9.99e-01(6.35e-02)	6.52e-01(6.86e-02)
F13	3.37e-01(2.33e-02)	3.69e-01(3.01e-02)+	3.59e-01(2.77e-02)	3.45e-01(2.84e-02)
F14	3.04e-01(1.32e-02)	3.05e-01(2.10e-02)+	3.01e-01(1.71e-02)	2.95e-01(1.93e-02)
F15	3.39e+01(2.49e+00)	3.29e+01(1.78e+00)+	3.03e+01(2.11e+00)	1.95e+01(1.27e+00)
F16	4.20e+01(3.58e-01)	4.16e+01(3.62e-01)+	4.18e+01(3.43e-01)	4.00e+01(5.78e-01)
F17	2.54e+04(1.32e+04)	1.18e+04(5.27e+03)	1.29e+04(5.05e+03)	1.30e+04(5.76e+03)
F18	3.69e+02(1.32e+02)	4.92e+02(2.35e+02)	4.11e+02(2.51e+02)	4.14e+02(1.65e+02)
F19	9.71e+01(3.46e+00)	9.39e+01(1.61e+00)	9.52e+01(3.27e+00)	7.94e+01(2.74e+01)
F20	8.90e+03(1.82e+04)	1.07e+04(1.90e+04)	5.92e+03(1.50e+04)	5.81e+02(1.01e+03)
F21	3.25e+03(1.38e+03)	2.57e+03(5.94e+02)	2.44e+03(5.75e+02)	2.64e+03(6.90e+02)
F22	2.25e+03(1.55e+02)	2.20e+03(2.67e+02)	2.12e+03(2.37e+02)	1.18e+03(1.71e+02)
F23	3.48e+02(4.44e-10)	3.48e+02(1.08e-13)	3.48e+02(1.74e-13)	3.48e+02(1.86e-13)
F24	3.91e+02(2.71e+00)	3.89e+02(2.94e+00)	3.89e+02(2.70e+00)	3.79e+02(3.45e+00)
F25	2.01e+02(5.15e+00)	2.35e+02(1.81e+01)	2.17e+02(2.09e+01)	2.22e+02(2.13e+01)
F26	2.00e+02(4.85e-03)	2.00e+02(4.40e-03)	2.00e+02(4.28e-03)	2.00e+02(3.59e-03)
F27	5.53e+02(3.83e+01)	4.89e+02(4.81e+01)	5.17e+02(4.10e+01)	4.63e+02(6.00e+01)
F28	2.30e+03(1.00e+02)	2.14e+03(1.68e+02)	2.25e+03(8.94e+01)	2.21e+03(6.07e+01)
F29	1.39e+03(1.30e+02)	8.56e+02(1.28e+02)	8.80e+02(1.24e+02)	8.95e+02(1.78e+02)
F30	7.18e+03(7.61e+02)	7.32e+03(1.01e+03)	7.56e+03(1.18e+03)	7.54e+03(1.09e+03)
rank	4	3	2	1
