# Supplementary material for: A novel differential evolution algorithm with multi-population and elites regeneration
Source: PLoS One. 2024 Apr 25;19(4):e0302207. doi: 10.1371/journal.pone.0302207 (PMC11045134; doi:10.1371/journal.pone.0302207)
Supplement: S4 Table — (PDF) [file pone.0302207.s004.pdf]

D=30	**0.001**	**0.005**	**0.01**	**0.05**
Fi	**Mean(St.D)**	**Mean(St.D)**	**Mean(St.D)**	**Mean(St.D)**
F1	5.75e+02(9.28e+02)	7.66e+02(1.09e+03)	9.54e+02(1.61e+03)	1.26e+03(1.94e+03)
F2	2.83e-12(1.98e-11)	0.00e+00(0.00e+00)	1.13e-13(7.89e-13)	0.00e+00(0.00e+00)
F3	7.23e+00(9.95e+00)	7.16e-01(1.26e+00)	6.21e+00(1.11e+01)	2.46e+00(3.92e+00)
F4	1.45e-28(1.64e-28)	1.55e-28(1.67e-28)	2.94e-28(7.55e-28)	1.20e-28(2.88e-28)
F5	2.00e+01(2.74e-04)	2.00e+01(1.71e-04)	2.00e+01(7.98e-04)	2.01e+01(1.70e-02)
F6	9.80e+00(1.60e+00)	9.58e+00(2.19e+00)	1.00e+01(2.12e+00)	9.76e+00(2.01e+00)
F7	1.48e-04(1.04e-03)	0.00e+00(0.00e+00)	4.44e-04(1.76e-03)	3.82e-10(2.67e-09)
F8	0.00e+00(0.00e+00)	0.00e+00(0.00e+00)	0.00e+00(0.00e+00)	0.00e+00(0.00e+00)
F9	2.14e+01(4.02e+00)	2.15e+01(4.38e+00)	2.27e+01(5.17e+00)	2.33e+01(4.29e+00)
F10	6.25e-03(1.04e-02)	4.58e-03(8.62e-03)	9.99e-03(1.12e-02)	5.83e-03(1.18e-02)
F11	6.25e-03(1.04e-02)	1.52e+03(2.08e+02)	1.49e+03(1.98e+02)	1.50e+03(1.91e+02)
F12	1.75e-01(3.41e-02)	1.64e-01(3.01e-02)	1.76e-01(3.66e-02)	1.86e-01(3.42e-02)
F13	1.98e-01(4.09e-02)	1.99e-01(3.10e-02)	2.07e-01(3.44e-02)	2.19e-01(3.56e-02)
F14	2.25e-01(3.00e-02)	2.28e-01(4.04e-02)	2.31e-01(3.52e-02)	2.38e-01(4.23e-02)
F15	2.23e+00(3.71e-01)	2.37e+00(3.71e-01)	2.30e+00(3.00e-01)	2.85e+00(3.78e-01)
F16	9.35e+00(3.32e-01)	9.28e+00(4.62e-01)	9.35e+00(4.08e-01)	9.43e+00(3.09e-01)
F17	1.27e+04(8.10e+04)	1.22e+03(4.22e+02)	1.33e+03(5.20e+02)	1.17e+03(3.78e+02)
F18	1.30e+02(2.68e+02)	8.49e+01(3.02e+01)	8.24e+01(3.33e+01)	8.74e+01(6.89e+01)
F19	4.81e+00(6.56e-01)	4.80e+00(7.44e-01)	5.01e+00(8.27e-01)	4.80e+00(7.08e-01)
F20	1.05e+03(1.98e+03)	1.17e+03(2.66e+03)	1.18e+03(2.22e+03)	6.50e+02(1.63e+03)
F21	1.09e+03(5.61e+03)	2.90e+02(2.90e+02)	4.51e+03(1.79e+04)	1.94e+03(9.93e+03)
F22	1.14e+02(7.44e+01)	1.27e+02(6.51e+01)	1.19e+02(6.68e+01)	1.27e+02(6.30e+01)
F23	2.90e+02(1.86e-13)	2.90e+02(1.89e-13)	2.90e+02(1.83e-13)	2.90e+02(1.87e-13)
F24	2.01e+02(7.28e-02)	2.01e+02(1.20e-01)	2.01e+02(5.95e-02)	2.01e+02(7.54e-02)
F25	2.09e+02(1.92e+00)	2.08e+02(1.48e+00)	2.09e+02(2.15e+00)	2.09e+02(2.07e+00)
F26	1.00e+02(3.66e-02)	1.00e+02(3.96e-02)	1.00e+02(3.69e-02)	1.00e+02(3.96e-02)
F27	3.80e+02(4.14e+01)	3.73e+02(4.19e+01)	3.83e+02(4.14e+01)	3.79e+02(3.96e+01)
F28	4.25e+02(9.83e+00)	4.22e+02(8.57e+00)	4.24e+02(8.48e+00)	4.23e+02(8.53e+00)
F29	1.03e+07(5.15e+06)	1.04e+07(4.95e+06)	1.10e+07(4.47e+06)	1.05e+07(4.92e+06)
F30	7.89e+02(2.12e+02)	7.55e+02(2.21e+02)	8.13e+02(2.11e+02)	7.72e+02(2.31e+02)
rank	2	1	4	3
