# Supplementary material for: A novel differential evolution algorithm with multi-population and elites regeneration
Source: PLoS One. 2024 Apr 25;19(4):e0302207. doi: 10.1371/journal.pone.0302207 (PMC11045134; doi:10.1371/journal.pone.0302207)
Supplement: S5 Table — (PDF) [file pone.0302207.s005.pdf]

D=50	**0.001**	**0.005**	**0.01**	**0.05**
Fi	**Mean(St.D)**	**Mean(St.D)**	**Mean(St.D)**	**Mean(St.D)**
F1	2.88e+04(1.55e+04)	2.32e+04(1.10e+04)	2.13e+04(1.12e+04)	3.33e+04(1.93e+04)
F2	3.20e-22(3.27e-22)	2.78e-22(2.41e-22)	2.43e-22(3.04e-22)	3.32e-22(3.16e-22)
F3	2.31e+03(2.43e+03)	2.69e+03(2.58e+03)	2.50e+03(2.35e+03)	2.31e+03(2.19e+03)
F4	1.96e+01(3.92e+01)	1.96e+01(3.92e+01)	1.37e+01(3.40e+01)	2.55e+01(4.30e+01)
F5	2.00e+01(9.91e-05)	2.00e+01(5.61e-04)	2.00e+01(1.72e-03)	2.03e+01(2.72e-02)
F6	2.38e+01(3.19e+00)	2.18e+01(6.80e+00)	2.17e+01(7.39e+00)	2.25e+01(6.14e+00)
F7	7.40e-04(2.57e-03)	0.00e+00(0.00e+00)	0.00e+00(0.00e+00)	6.12e-04(2.11e-03)
F8	5.96e-11(1.18e-10)	1.84e-11(2.34e-11)	1.46e-11(1.87e-11)	1.18e-11(2.48e-11)
F9	4.68e+01(8.86e+00)	4.48e+01(8.79e+00)	4.52e+01(7.43e+00)	4.85e+01(8.59e+00)
F10	4.57e+00(1.08e+00)	2.89e+00(1.01e+00)	2.47e+00(8.64e-01)	4.10e+00(8.24e-01)
F11	4.31e+03(3.05e+02)	3.93e+03(3.33e+02)	3.94e+03(3.44e+02)	3.98e+03(5.75e+02)
F12	3.04e-01(5.33e-02)	2.83e-01(4.91e-02)	2.96e-01(4.31e-02)	3.16e-01(4.29e-02)
F13	2.79e-01(3.18e-02)	2.95e-01(3.19e-02)	2.88e-01(3.69e-02)	2.98e-01(3.75e-02)
F14	2.81e-01(2.88e-02)	2.80e-01(2.71e-02)	2.82e-01(2.92e-02)	2.82e-01(2.44e-02)
F15	6.13e+00(6.25e-01)	5.89e+00(6.75e-01)	6.05e+00(6.54e-01)	7.16e+00(7.53e-01)
F16	1.80e+01(4.34e-01)	1.80e+01(4.07e-01)	1.80e+01(4.10e-01)	1.80e+01(4.35e-01)
F17	2.21e+03(4.49e+02)	2.00e+03(4.34e+02)	2.17e+03(5.24e+02)	2.10e+03(4.96e+02)
F18	1.33e+02(3.00e+01)	1.33e+02(3.00e+01)	1.32e+02(2.58e+01)	1.32e+02(3.15e+01)
F19	1.53e+01(8.76e+00)	1.68e+01(9.44e+00)	1.62e+01(9.34e+00)	1.63e+01(9.60e+00)
F20	3.11e+03(5.72e+03)	2.96e+03(5.29e+03)	1.89e+03(4.84e+03)	2.99e+03(5.19e+03)
F21	1.21e+04(7.67e+04)	1.15e+03(3.02e+02)	1.00e+03(3.06e+02)	1.18e+04(7.50e+04)
F22	3.67e+02(1.33e+02)	3.88e+02(1.19e+02)	3.58e+02(1.15e+02)	3.70e+02(1.49e+02)
F23	3.27e+02(3.38e-13)	3.27e+02(3.39e-13)	3.27e+02(3.26e-13)	3.27e+02(3.36e-13)
F24	2.03e+02(2.31e-01)	2.03e+02(1.73e-01)	2.03e+02(1.95e-01)	2.03e+02(1.90e-01)
F25	2.04e+02(2.80e+00)	2.04e+02(2.44e+00)	2.05e+02(1.79e+00)	2.05e+02(2.11e+00)
F26	1.00e+02(2.68e-02)	1.00e+02(3.29e-02)	1.00e+02(3.51e-02)	1.00e+02(2.84e-02)
F27	4.09e+02(1.07e+01)	4.10e+02(1.03e+01)	4.10e+02(9.76e+00)	4.09e+02(1.04e+01)
F28	5.01e+02(1.38e+01)	4.99e+02(1.58e+01)	4.71e+02(1.60e+01)	4.96e+02(1.37e+01)
F29	1.21e+03(1.59e+03)	1.29e+03(1.44e+03)	9.78e+02(3.96e+01)	1.05e+03(4.72e+02)
F30	4.42e+03(1.15e+03)	4.80e+03(1.13e+03)	4.38e+03(1.11e+03)	4.61e+03(1.01e+03)
rank	3	2	1	4
