# Supplementary material for: A novel differential evolution algorithm with multi-population and elites regeneration
Source: PLoS One. 2024 Apr 25;19(4):e0302207. doi: 10.1371/journal.pone.0302207 (PMC11045134; doi:10.1371/journal.pone.0302207)
Supplement: S6 Table — (PDF) [file pone.0302207.s006.pdf]

D=100	0.001	0.005	0.01	0.005
Fi	**Mean(St.D)**	**Mean(St.D)**	**Mean(St.D)**	**Mean(St.D)**
F1	1.90e+05(5.63e+04)	2.04e+05(4.76e+04)	1.86e+05(4.56e+04)	2.26e+05(7.04e+04)
F2	6.49e-19(3.69e-18)	3.42e-19(1.89e-18)	5.02e-19(1.70e-18)	1.13e-18(6.08e-18)
F3	5.70e+03(5.01e+03)	5.02e+03(4.85e+03)	3.73e+03(4.86e+03)	4.54e+03(4.77e+03)
F4	9.26e+01(4.26e+01)	8.59e+01(4.59e+01)	9.14e+01(4.59e+01)	8.32e+01(3.47e+01)
F5	2.00e+01(5.84e-03)	2.00e+01(4.02e-03)	2.01e+01(6.60e-03)	2.06e+01(2.80e-02)
F6	6.32e+01(2.48e+01)	6.59e+01(2.60e+01)	7.00e+01(2.10e+01)	7.33e+01(1.75e+01)
F7	9.36e-04(3.44e-03)	1.97e-04(1.38e-03)	1.08e-03(3.94e-03)	3.55e-17(6.45e-17)
F8	1.16e+01(1.79e+00)	8.12e+00(1.65e+00)	7.47e+00(1.25e+00)	7.13e+00(1.37e+00)
F9	1.72e+02(1.93e+01)	1.20e+02(1.61e+01)	1.16e+02(1.24e+01)	1.14e+02(1.38e+01)
F10	6.41e+02(1.03e+02)	2.85e+02(7.62e+01)	2.46e+02(8.33e+01)	2.51e+02(8.11e+01)
F11	1.43e+04(5.02e+02)	1.20e+04(5.95e+02)	1.18e+04(5.18e+02)	1.16e+04(6.30e+02)
F12	6.15e-01(5.19e-02)	6.54e-01(6.60e-02)	6.88e-01(5.93e-02)	6.52e-01(6.86e-02)
F13	3.37e-01(3.04e-02)	3.48e-01(2.24e-02)	3.46e-01(2.65e-02)	3.45e-01(2.84e-02)
F14	2.99e-01(2.02e-02)	3.02e-01(1.78e-02)	2.97e-01(1.72e-02)	2.95e-01(1.93e-02)
F15	2.22e+01(1.89e+00)	2.22e+01(2.38e+00)	2.24e+01(1.91e+00)	1.95e+01(1.27e+00)
F16	4.07e+01(4.85e-01)	4.07e+01(5.67e-01)	4.09e+01(4.47e-01)	4.00e+01(5.78e-01)
F17	1.14e+04(5.48e+03)	1.15e+04(3.01e+03)	1.19e+04(3.53e+03)	1.30e+04(5.76e+03)
F18	4.33e+02(2.30e+02)	4.64e+02(2.59e+02)	4.30e+02(2.66e+02)	4.14e+02(1.65e+02)
F19	9.49e+01(1.96e+00)	9.46e+01(6.31e+00)	9.56e+01(3.66e+00)	7.94e+01(2.74e+01)
F20	2.08e+03(8.22e+03)	3.18e+03(1.12e+04)	3.17e+03(1.10e+04)	5.81e+02(1.01e+03)
F21	2.41e+03(5.82e+02)	2.59e+03(5.35e+02)	2.66e+03(6.65e+02)	2.64e+03(6.90e+02)
F22	1.60e+03(1.68e+02)	1.26e+03(2.16e+02)	1.32e+03(2.01e+02)	1.18e+03(1.71e+02)
F23	3.48e+02(1.63e-13)	3.48e+02(1.66e-13)	3.48e+02(1.64e-13)	3.48e+02(1.86e-13)
F24	3.86e+02(3.01e+00)	3.79e+02(3.59e+00)	3.76e+02(3.77e+00)	3.79e+02(3.45e+00)
F25	2.21e+02(2.17e+01)	2.23e+02(2.12e+01)	2.21e+02(2.07e+01)	2.22e+02(2.13e+01)
F26	2.00e+02(3.80e-03)	2.00e+02(4.32e-03)	2.00e+02(4.51e-03)	2.00e+02(3.59e-03)
F27	4.96e+02(3.46e+01)	5.15e+02(4.69e+01)	5.08e+02(4.96e+01)	4.63e+02(6.00e+01)
F28	2.22e+03(6.33e+01)	2.19e+03(9.56e+01)	2.18e+03(7.82e+01)	2.21e+03(6.07e+01)
F29	8.66e+02(1.23e+02)	8.75e+02(1.30e+02)	8.81e+02(1.20e+02)	8.95e+02(1.78e+02)
F30	7.44e+03(1.02e+03)	7.50e+03(9.66e+02)	7.46e+03(1.23e+03)	7.54e+03(1.09e+03)
rank	3	4	2	1
