# Supplementary material for: A novel differential evolution algorithm with multi-population and elites regeneration
Source: PLoS One. 2024 Apr 25;19(4):e0302207. doi: 10.1371/journal.pone.0302207 (PMC11045134; doi:10.1371/journal.pone.0302207)
Supplement: S7 Table — (PDF) [file pone.0302207.s007.pdf]

D=30\NP	**50**	**150**	**200**	**300**	**400**	**STD**
Fi	**Mean(St.D)**	**Mean(St.D)**	**Mean(St.D)**	**Mean(St.D)**	**Mean(St.D)**	**Mean(St.D)**
F1	4.97e+02(4.34e+02)	1.69e+01(4.64e+01)	6.15e-10(8.19e-10)	1.21e-16(2.70e-16)	1.66e-16(3.55e-16)	7.66e+02(1.09e+03)
F2	0.00e+00(0.00e+00)	3.04e-12(9.11e-12)	0.00e+00(0.00e+00)	0.00e+00(0.00e+00)	9.73e-21(2.87e-20)	0.00e+00(0.00e+00)
F3	1.54e-02(3.69e-02)	1.02e+01(1.04e+01)	2.23e+01(2.87e+01)	3.36e+01(3.90e+01)	3.12e+01(2.17e+01)	7.16e-01(1.26e+00)
F4	1.98e-15(3.93e-15)	3.14e-29(2.25e-29)	3.05e-29(2.38e-29)	8.28e-19(1.74e-18)	1.86e-06(4.36e-06)	1.55e-28(1.67e-28)
F5	2.00e+01(2.06e-04)	2.00e+01(1.93e-04)	2.00e+01(3.31e-04)	2.00e+01(5.51e-04)	2.00e+01(1.80e-03)	2.00e+01(1.71e-04)
F6	7.56e+00(3.25e+00)	1.14e+01(1.93e+00)	1.27e+01(1.67e+00)	1.35e+01(6.89e-01)	1.49e+01(1.24e+00)	9.58e+00(2.19e+00)
F7	3.45e-03(5.42e-03)	0.00e+00(0.00e+00)	0.00e+00(0.00e+00)	0.00e+00(0.00e+00)	0.00e+00(0.00e+00)	0.00e+00(0.00e+00)
F8	0.00e+00(0.00e+00)	0.00e+00(0.00e+00)	1.53e-10(9.51e-11)	3.21e-04(1.02e-04)	2.26e-01(7.78e-02)	0.00e+00(0.00e+00)
F9	2.60e+01(4.28e+00)	2.13e+01(6.68e+00)	2.29e+01(2.34e+00)	3.21e+01(6.13e+00)	3.85e+01(6.20e+00)	2.15e+01(4.38e+00)
F10	7.49e-02(2.97e-02)	3.14e-02(1.98e-02)	5.54e-01(1.57e-01)	1.08e+01(2.06e+00)	3.84e+01(8.36e+00)	4.58e-03(8.62e-03)
F11	1.57e+03(2.36e+02)	1.69e+03(1.77e+02)	1.73e+03(1.86e+02)	2.11e+03(2.26e+02)	2.45e+03(2.06e+02)	1.52e+03(2.08e+02)
F12	1.17e-01(2.18e-02)	2.07e-01(3.36e-02)	2.03e-01(3.57e-02)	2.78e-01(5.21e-02)	3.23e-01(5.32e-02)	1.64e-01(3.01e-02)
F13	2.15e-01(4.54e-02)	1.87e-01(3.39e-02)	2.02e-01(2.32e-02)	1.93e-01(1.82e-02)	1.96e-01(2.70e-02)	1.99e-01(3.10e-02)
F14	2.28e-01(3.54e-02)	2.23e-01(1.58e-02)	2.08e-01(2.87e-02)	2.04e-01(1.61e-02)	2.01e-01(3.01e-02)	2.28e-01(4.04e-02)
F15	2.33e+00(3.74e-01)	2.54e+00(2.01e-01)	2.72e+00(2.03e-01)	2.93e+00(5.08e-01)	3.50e+00(4.24e-01)	2.37e+00(3.71e-01)
F16	9.49e+00(3.14e-01)	9.41e+00(2.86e-01)	9.67e+00(2.86e-01)	9.72e+00(3.32e-01)	9.95e+00(2.33e-01)	9.28e+00(4.62e-01)
F17	1.29e+03(2.10e+02)	9.26e+02(2.05e+02)	7.52e+02(3.19e+02)	4.94e+02(3.06e+02)	6.04e+02(1.68e+02)	1.22e+03(4.22e+02)
F18	1.58e+02(4.96e+01)	5.20e+01(3.11e+01)	4.47e+01(2.36e+01)	2.03e+01(9.03e+00)	1.80e+01(7.41e+00)	8.49e+01(3.02e+01)
F19	5.23e+00(8.81e-01)	5.10e+00(7.81e-01)	4.72e+00(5.12e-01)	4.85e+00(9.32e-01)	4.91e+00(8.08e-01)	4.80e+00(7.44e-01)
F20	8.19e+01(7.71e+01)	1.34e+03(2.45e+03)	1.26e+03(1.42e+03)	1.37e+03(1.25e+03)	1.79e+03(1.56e+03)	1.17e+03(2.66e+03)
F21	6.24e+02(2.40e+02)	1.92e+03(5.01e+03)	5.42e+03(1.56e+04)	5.73e+03(1.64e+04)	4.93e+03(1.36e+04)	2.90e+02(2.90e+02)
F22	1.61e+02(6.18e+01)	1.29e+02(6.63e+01)	6.48e+01(5.10e+01)	9.76e+01(5.15e+01)	1.10e+02(4.88e+01)	1.27e+02(6.51e+01)
F23	2.90e+02(3.60e-14)	2.90e+02(6.48e-14)	2.90e+02(1.80e-14)	2.90e+02(5.68e-14)	2.90e+02(5.68e-14)	2.90e+02(1.89e-13)
F24	2.01e+02(5.88e-02)	2.01e+02(7.89e-02)	2.01e+02(6.39e-02)	2.01e+02(6.80e-02)	2.01e+02(4.88e-02)	2.01e+02(1.20e-01)
F25	2.11e+02(1.95e+00)	2.07e+02(2.49e+00)	2.07e+02(2.50e+00)	2.08e+02(1.17e+00)	2.07e+02(1.05e+00)	2.08e+02(1.48e+00)
F26	1.00e+02(4.94e-02)	1.00e+02(3.25e-02)	1.00e+02(3.00e-02)	1.00e+02(2.26e-02)	1.00e+02(2.93e-02)	1.00e+02(3.96e-02)
F27	4.14e+02(3.76e+01)	3.79e+02(3.98e+01)	3.93e+02(3.12e+01)	3.82e+02(4.11e+01)	3.93e+02(3.13e+01)	3.73e+02(4.19e+01)
F28	4.45e+02(9.65e+00)	4.19e+02(5.03e+00)	4.13e+02(3.53e+00)	4.11e+02(2.20e+00)	4.12e+02(2.47e+00)	4.22e+02(8.57e+00)
F29	1.22e+07(4.23e+06)	1.01e+07(5.07e+06)	1.28e+07(1.93e+05)	1.02e+07(5.09e+06)	1.02e+07(5.11e+06)	1.04e+07(4.95e+06)
F30	9.86e+02(3.45e+02)	8.13e+02(1.25e+02)	7.29e+02(2.46e+02)	5.81e+02(1.28e+02)	5.75e+02(1.38e+02)	7.55e+02(2.21e+02)
+/=/−	17/7/6	14/6/10	14/6/10	14/7/9	15/5/10	−/−/−
