# Supplementary material for: A novel differential evolution algorithm with multi-population and elites regeneration
Source: PLoS One. 2024 Apr 25;19(4):e0302207. doi: 10.1371/journal.pone.0302207 (PMC11045134; doi:10.1371/journal.pone.0302207)
Supplement: S8 Table — (PDF) [file pone.0302207.s008.pdf]

D=50\NP	50	100	150	300	400	STD
Fi	**Mean(St.D)**	**Mean(St.D)**	**Mean(St.D)**	**Mean(St.D)**	**Mean(St.D)**	**Mean(St.D)**
F1	6.65e+03(1.06e+04)	1.12e+04(5.49e+03)	2.11e+04(1.10e+04)	2.23e+04(1.27e+04)	1.37e+04(9.48e+03)	2.13e+04(1.12e+04)
F2	3.54e-17(8.01e-17)	4.42e-21(1.08e-20)	3.53e-16(2.47e-15)	3.86e-22(1.94e-22)	1.47e-20(8.89e-20)	2.43e-22(3.04e-22)
F3	1.74e+03(2.95e+03)	2.03e+03(2.34e+03)	2.25e+03(2.44e+03)	2.41e+03(2.00e+03)	2.30e+03(1.83e+03)	2.50e+03(2.35e+03)
F4	7.15e+00(2.38e+01)	1.97e+01(3.92e+01)	1.19e+01(3.19e+01)	2.75e+01(4.40e+01)	2.58e+01(4.16e+01)	1.37e+01(3.40e+01)
F5	2.00e+01(7.91e-04)	2.00e+01(1.19e-03)	2.00e+01(1.63e-03)	2.00e+01(2.81e-03)	2.00e+01(4.12e-03)	2.00e+01(1.72e-03)
F6	1.98e+01(4.13e+00)	1.58e+01(8.21e+00)	2.09e+01(6.44e+00)	2.57e+01(5.38e+00)	2.96e+01(2.21e+00)	2.17e+01(7.39e+00)
F7	5.62e-03(6.40e-03)	3.06e-03(4.34e-03)	8.38e-04(2.54e-03)	0.00e+00(0.00e+00)	0.00e+00(0.00e+00)	0.00e+00(0.00e+00)
F8	1.78e-16(8.14e-16)	0.00e+00(0.00e+00)	0.00e+00(0.00e+00)	1.12e-03(4.42e-04)	8.85e-01(2.46e-01)	1.46e-11(1.87e-11)
F9	6.33e+01(1.14e+01)	4.80e+01(8.07e+00)	4.40e+01(6.79e+00)	4.81e+01(8.31e+00)	5.35e+01(7.80e+00)	4.52e+01(7.43e+00)
F10	6.10e-02(3.03e-02)	9.75e-03(1.30e-02)	2.37e-01(3.58e-02)	1.51e+01(2.78e+00)	5.43e+01(1.14e+01)	2.47e+00(8.64e-01)
F11	3.75e+03(3.50e+02)	3.59e+03(3.33e+02)	3.54e+03(3.67e+02)	4.08e+03(4.28e+02)	4.52e+03(3.58e+02)	3.94e+03(3.44e+02)
F12	1.36e-01(2.11e-02)	1.84e-01(3.68e-02)	2.52e-01(4.45e-02)	3.42e-01(5.89e-02)	3.82e-01(5.18e-02)	2.96e-01(4.31e-02)
F13	3.68e-01(7.23e-02)	3.12e-01(4.47e-02)	3.02e-01(4.27e-02)	2.85e-01(2.84e-02)	2.87e-01(2.64e-02)	2.88e-01(3.69e-02)
F14	3.20e-01(7.96e-02)	3.16e-01(8.31e-02)	2.93e-01(4.17e-02)	2.71e-01(2.67e-02)	2.77e-01(2.22e-02)	2.82e-01(2.92e-02)
F15	1.16e+01(2.87e+00)	6.22e+00(8.44e-01)	5.65e+00(8.33e-01)	6.66e+00(7.57e-01)	7.55e+00(8.64e-01)	6.05e+00(6.54e-01)
F16	1.79e+01(3.91e-01)	1.76e+01(4.70e-01)	1.78e+01(4.29e-01)	1.83e+01(3.06e-01)	1.85e+01(2.99e-01)	1.80e+01(4.10e-01)
F17	2.66e+03(9.66e+02)	2.51e+03(5.85e+02)	2.27e+03(5.91e+02)	1.92e+03(4.76e+02)	1.95e+03(4.74e+02)	2.17e+03(5.24e+02)
F18	3.17e+02(3.90e+02)	1.54e+02(3.50e+01)	1.45e+02(8.02e+01)	1.20e+02(2.75e+01)	1.07e+02(2.48e+01)	1.32e+02(2.58e+01)
F19	1.50e+01(4.47e+00)	1.38e+01(7.89e+00)	1.63e+01(8.66e+00)	1.61e+01(9.58e+00)	1.36e+01(7.23e+00)	1.62e+01(9.34e+00)
F20	2.45e+03(5.12e+03)	1.54e+03(3.47e+03)	3.03e+03(5.59e+03)	3.44e+03(5.33e+03)	2.26e+03(4.29e+03)	1.89e+03(4.84e+03)
F21	1.24e+04(7.59e+04)	2.66e+04(1.25e+05)	1.18e+03(3.43e+02)	8.69e+02(2.50e+02)	8.35e+02(2.26e+02)	1.00e+03(3.06e+02)
F22	5.02e+02(1.91e+02)	3.91e+02(1.13e+02)	3.65e+02(1.23e+02)	3.52e+02(9.58e+01)	3.46e+02(1.15e+02)	3.58e+02(1.15e+02)
F23	3.27e+02(2.59e-13)	3.27e+02(3.17e-13)	3.27e+02(3.33e-13)	3.27e+02(3.40e-13)	3.27e+02(3.41e-13)	3.27e+02(3.26e-13)
F24	2.03e+02(3.83e-01)	2.03e+02(2.37e-01)	2.03e+02(2.01e-01)	2.03e+02(1.54e-01)	2.03e+02(1.73e-01)	2.03e+02(1.95e-01)
F25	2.07e+02(1.49e+00)	2.05e+02(2.53e+00)	2.05e+02(2.19e+00)	2.03e+02(2.80e+00)	2.03e+02(2.60e+00)	2.05e+02(1.79e+00)
F26	1.00e+02(6.01e-02)	1.00e+02(4.07e-02)	1.00e+02(3.41e-02)	1.00e+02(2.27e-02)	1.00e+02(2.96e-02)	1.00e+02(3.51e-02)
F27	4.22e+02(5.04e+00)	4.19e+02(7.64e+00)	4.12e+02(1.03e+01)	4.03e+02(7.36e+00)	4.02e+02(5.51e+00)	4.10e+02(9.76e+00)
F28	6.22e+02(6.34e+01)	5.38e+02(3.53e+01)	5.10e+02(2.28e+01)	4.84e+02(1.17e+01)	4.81e+02(1.11e+01)	4.71e+02(1.60e+01)
F29	1.48e+03(1.52e+03)	1.24e+06(8.68e+06)	1.16e+03(7.07e+02)	9.69e+02(2.01e+01)	9.68e+02(1.38e+01)	9.78e+02(3.96e+01)
F30	6.27e+03(1.63e+03)	5.03e+03(1.12e+03)	4.94e+03(1.36e+03)	4.45e+03(9.49e+02)	4.67e+03(8.57e+02)	4.38e+03(1.11e+03)
+/=/−	16/4/10	15/5/10	14/5/11	14/5/11	13/5/12	−/−/−
