# Supplementary material for: A novel differential evolution algorithm with multi-population and elites regeneration
Source: PLoS One. 2024 Apr 25;19(4):e0302207. doi: 10.1371/journal.pone.0302207 (PMC11045134; doi:10.1371/journal.pone.0302207)
Supplement: S9 Table — (PDF) [file pone.0302207.s009.pdf]

D=100\NP	**50**	**100**	**150**	**200**	**300**	**400**
Fi	**Mean(St.D)**	**Mean(St.D)**	**Mean(St.D)**	**Mean(St.D)**	**Mean(St.D)**	**Mean(St.D)**
F1	9.80e+04(4.88e+04)	9.68e+04(4.20e+04)	1.38e+05(5.82e+04)	1.56e+05(5.30e+04)	1.92e+05(6.48e+04)	2.26e+05(7.04e+04)
F2	2.12e-05(7.98e-05)	5.29e-14(1.83e-13)	3.34e-17(1.14e-16)	7.09e-19(3.28e-18)	2.49e-20(3.85e-20)	1.13e-18(6.08e-18)
F3	1.32e+03(2.16e+03)	1.85e+03(3.12e+03)	3.03e+03(4.29e+03)	3.29e+03(4.28e+03)	4.30e+03(4.65e+03)	4.54e+03(4.77e+03)
F4	1.02e+02(4.13e+01)	5.66e+01(4.29e+01)	5.51e+01(4.88e+01)	5.49e+01(5.40e+01)	7.55e+01(5.41e+01)	8.32e+01(3.47e+01)
F5	2.02e+01(4.10e-02)	2.04e+01(6.02e-02)	2.04e+01(6.85e-02)	2.05e+01(3.41e-02)	2.05e+01(3.35e-02)	2.06e+01(2.80e-02)
F6	6.32e+01(8.94e+00)	5.76e+01(1.26e+01)	5.61e+01(1.83e+01)	6.10e+01(1.96e+01)	6.49e+01(2.07e+01)	7.33e+01(1.75e+01)
F7	1.53e-03(3.97e-03)	8.36e-04(4.27e-03)	1.97e-03(5.61e-03)	1.48e-03(3.82e-03)	1.51e-16(1.70e-16)	3.55e-17(6.45e-17)
F8	1.99e-02(1.39e-01)	1.88e-15(1.64e-15)	4.26e-16(7.59e-16)	1.07e-16(4.22e-16)	6.68e-03(4.72e-03)	7.13e+00(1.37e+00)
F9	2.19e+02(3.93e+01)	1.47e+02(1.75e+01)	1.28e+02(1.72e+01)	1.19e+02(1.74e+01)	1.12e+02(1.07e+01)	1.14e+02(1.38e+01)
F10	4.99e+00(2.32e+01)	1.68e-02(9.27e-03)	2.55e+00(5.06e-01)	1.50e+01(1.57e+00)	4.55e+01(6.31e+00)	2.51e+02(8.11e+01)
F11	1.04e+04(6.52e+02)	1.00e+04(5.17e+02)	1.01e+04(6.97e+02)	1.09e+04(9.53e+02)	1.15e+04(7.25e+02)	1.16e+04(6.30e+02)
F12	2.49e-01(3.13e-02)	3.32e-01(3.17e-02)	4.13e-01(4.21e-02)	5.14e-01(5.18e-02)	6.38e-01(7.69e-02)	6.52e-01(6.86e-02)
F13	4.72e-01(6.04e-02)	3.90e-01(4.11e-02)	3.73e-01(3.34e-02)	3.64e-01(3.16e-02)	3.56e-01(2.94e-02)	3.45e-01(2.84e-02)
F14	3.14e-01(2.81e-02)	3.15e-01(2.60e-02)	3.04e-01(2.25e-02)	3.07e-01(2.13e-02)	3.05e-01(1.81e-02)	2.95e-01(1.93e-02)
F15	5.51e+01(1.06e+01)	3.41e+01(5.07e+00)	2.94e+01(3.69e+00)	2.80e+01(3.52e+00)	2.71e+01(2.23e+00)	1.95e+01(1.27e+00)
F16	4.04e+01(6.21e-01)	4.04e+01(4.73e-01)	4.02e+01(4.44e-01)	4.02e+01(5.62e-01)	4.04e+01(4.39e-01)	4.00e+01(5.78e-01)
F17	3.00e+04(1.38e+04)	1.31e+04(5.95e+03)	1.24e+04(4.37e+03)	1.40e+04(4.97e+03)	1.42e+04(5.48e+03)	1.30e+04(5.76e+03)
F18	1.37e+03(1.35e+03)	6.07e+02(5.41e+02)	7.55e+02(4.80e+02)	5.87e+02(4.40e+02)	4.49e+02(2.33e+02)	4.14e+02(1.65e+02)
F19	9.71e+01(2.33e+01)	9.49e+01(1.83e+01)	1.01e+02(9.65e+00)	1.00e+02(8.39e+00)	9.73e+01(4.97e+00)	7.94e+01(2.74e+01)
F20	1.20e+03(3.44e+03)	1.62e+03(5.77e+03)	5.40e+02(1.47e+02)	3.41e+03(1.13e+04)	3.13e+03(1.08e+04)	5.81e+02(1.01e+03)
F21	1.08e+04(7.68e+03)	4.23e+03(1.55e+03)	3.82e+03(1.29e+03)	3.81e+03(1.24e+03)	3.16e+03(9.50e+02)	2.64e+03(6.90e+02)
F22	1.53e+03(2.30e+02)	1.39e+03(2.40e+02)	1.25e+03(2.09e+02)	1.26e+03(2.05e+02)	1.35e+03(2.63e+02)	1.18e+03(1.71e+02)
F23	3.48e+02(7.45e-13)	3.48e+02(2.23e-13)	3.48e+02(2.17e-13)	3.48e+02(1.25e-13)	3.48e+02(1.57e-13)	3.48e+02(1.86e-13)
F24	3.81e+02(6.98e+00)	3.81e+02(5.05e+00)	3.79e+02(4.44e+00)	3.78e+02(4.34e+00)	3.78e+02(4.37e+00)	3.79e+02(3.45e+00)
F25	2.94e+02(1.31e+01)	2.71e+02(1.37e+01)	2.57e+02(1.38e+01)	2.47e+02(1.74e+01)	2.29e+02(2.18e+01)	2.22e+02(2.13e+01)
F26	2.00e+02(1.15e-02)	2.00e+02(4.16e-03)	2.00e+02(5.53e-03)	2.00e+02(4.28e-03)	2.00e+02(4.11e-03)	2.00e+02(3.59e-03)
F27	1.71e+03(2.02e+02)	1.10e+03(1.44e+02)	8.64e+02(9.72e+01)	7.28e+02(8.05e+01)	5.79e+02(6.76e+01)	4.63e+02(6.00e+01)
F28	3.02e+03(5.86e+02)	2.34e+03(1.48e+02)	2.25e+03(8.27e+01)	2.27e+03(2.27e+02)	2.19e+03(1.42e+02)	2.21e+03(6.07e+01)
F29	1.44e+03(3.05e+02)	1.36e+03(1.29e+02)	1.28e+03(2.04e+02)	1.21e+03(2.21e+02)	9.57e+02(1.88e+02)	8.95e+02(1.78e+02)
F30	8.88e+03(1.15e+03)	8.18e+03(1.63e+03)	8.02e+03(1.42e+03)	7.72e+03(1.11e+03)	7.34e+03(1.06e+03)	7.54e+03(1.09e+03)
+/=/−	20/2/8	19/4/7	16/4/10	17/3/10	14/2/14	−/−/−
