# Supplementary material for: A novel differential evolution algorithm with multi-population and elites regeneration
Source: PLoS One. 2024 Apr 25;19(4):e0302207. doi: 10.1371/journal.pone.0302207 (PMC11045134; doi:10.1371/journal.pone.0302207)
Supplement: S10 Table — (PDF) [file pone.0302207.s010.pdf]

D=30	**jDE**	**SaJADE**	**CoDE**	**EPSDE**	**SHADE**	**L-SHADE**	**EBJADE**
Fi	**Mean(St.D)**	**Mean(St.D)**	**Mean(St.D)**	**Mean(St.D)**	**Mean(St.D)**	**Mean(St.D)**	**Mean(St.D)**
F1	6.09e+05(3.85e+05)+	1.25e+03(2.56e+03)+	5.75e+04(3.73e+04)+	1.22e+04(8.88e+03)+	7.82e+02(1.53e+03)=	**1.30e-21**(3.80e-21)−	7.66e+02(1.09e+03)
F2	3.11e−16(3.10e−16)+	4.44e−24(1.51e−23)+	1.57e−06(1.50e−06)+	2.51e−22(4.16e−22)+	**0.00e+00**(0.00e+00)=	**0.00e+00**(0.00e+00)=	**0.00e+00**(0.00e+00)
F3	5.98e−21(5.43e−21)−	8.81e−01(1.30e+00)+	1.51e−09(1.07e−09)−	**0.00e+00**(0.00e+00)−	4.73e−28(1.60e−27)−	**0.00e+00**(0.00e+00)−	7.16e−01(1.26e+00)
F4	1.33e+01(1.33e+01)+	1.12e−28(2.09e−28)=	1.80e+01(2.83e+01)+	1.40e+00(8.87e+00)+	7.97e−02(5.58e−01)+	2.05e-15(1.42e-14)+	**1.55e−28**(1.67e−28)
F5	2.06e+01(4.74e−02)+	2.03e+01(3.52e−02)+	2.05e+01(5.58e−02)+	2.05e+01(4.75e−02)+	2.02e+01(2.73e−02)+	2.02e+01(4.34e-02)+	**2.00e+01**(1.71e−04)
F6	**9.50e−01**(2.29e+00)−	7.93e+00(3.66e+00)−	2.04e+01(1.30e+00)+	7.29e+00(7.04e+00)−	1.18e+00(2.69e+00)−	**0.00e+00**(0.00e+00)−	9.58e+00(2.19e+00)
F7	8.67e−21(1.99e−20)+	0.00e+00(0.00e+00)=	1.08e−03(3.12e−03)+	1.87e−03(4.55e−03)+	**0.00e+00**(0.00e+00)=	**0.00e+00**(0.00e+00)=	**0.00e+00**(0.00e+00)
F8	1.12e+00(2.40e+00)+	0.00e+00(0.00e+00)=	2.32e−13(2.10e−13)+	**0.00e+00**(0.00e+00)=	**0.00e+00**(0.00e+00)=	2.74e-15(3.50e-15)+	**0.00e+00**(0.00e+00)
F9	1.05e+02(9.37e+00)+	2.26e+01(3.60e+00)+	1.25e+02(9.27e+00)+	8.41e+01(8.60e+00)+	1.86e+01(3.11e+00)−	**9.26e+00**(1.69e+00)−	2.15e+01(4.38e+00)
F10	1.30e+02(2.73e+01)+	**2.91e−03**(8.34e−03)−	2.42e+01(6.68e+00)+	1.59e+01(1.11e+01)+	5.00e−03(9.82e−03)+	7.21e-02(3.83e-02)+	4.58e−03(8.62e−03)
F11	4.66e+03(2.56e+02)+	1.72e+03(2.44e+02)+	4.30e+03(3.18e+02)+	3.94e+03(2.95e+02)+	1.56e+03(2.29e+02)+	1.56e+03(2.29e+02)+	**1.52e+03**(2.08e+02)
F12	9.86e−01(1.31e−01)+	3.04e−01(4.70e−02)+	8.44e−01(1.21e−01)+	7.94e−01(9.71e−02)+	2.01e−01(2.83e−02)+	2.54e-01(2.82e-02)+	**1.64e−01**(3.01e−02)
F13	3.17e−01(4.16e−02)+	1.89e−01(3.33e−02)=	4.92e−01(5.04e−02)+	2.82e−01(4.40e−02)+	1.94e−01(3.04e−02)=	**1.28e-01**(1.75e-02)−	1.99e−01(3.10e−02)
F14	2.70e−01(2.61e−02)+	**2.14e−01**(3.20e−02)=	2.83e−01(3.81e−02)+	2.56e−01(3.94e−02)+	2.33e−01(3.43e−02)+	2.30e-01(3.07e-02)=	2.28e−01(4.04e−02)
F15	1.07e+01(8.45e−01)+	3.11e+00(3.77e−01)+	1.44e+01(1.25e+00)+	8.62e+00(8.91e−01)+	2.95e+00(3.31e−01)+	2.61e+00(3.07e-01)+	**2.37e+00**(3.71e−01)
F16	1.14e+01(2.75e−001)+	9.31e+00(3.80e−01)=	1.13e+01(2.76e−01)+	1.08e+01(2.89e−01)+	9.37e+00(3.12e−01)+	9.29e+00(4.14e-01)+	**9.28e+00**(4.62e−01)
F17	2.12e+03(7.92e+02)+	1.26e+04(8.15e+04)=	1.33e+03(2.53e+02)=	1.25e+03(5.10e+02)=	1.17e+03(3.10e+02)=	**2.24e+02**(1.18e+02)−	1.22e+03(4.22e+02)
F18	4.49e+01(7.59e+00)−	5.05e+01(2.20e+01)−	4.28e+01(1.09e+01)−	8.46e+01(3.86e+01)=	5.22e+01(2.61e+01)−	**7.74e+00**(3.28e+00)−	8.49e+01(3.02e+01)
F19	1.25e+01(1.65e+00)+	4.15e+00(7.06e−01)−	7.23e+00(1.07e+00)+	4.71e+00(1.10e+00)=	4.25e+00(5.91e−01)−	**3.96e+00**(6.70e-01)−	4.80e+00(7.44e−01)
F20	5.62e+01(1.11e+01)−	2.53e+03(2.89e+03)+	3.63e+01(5.97e+00)−	2.66e+01(1.13e+01)−	9.24e+00(3.11e+00)−	**2.75e+00**(1.24e+00)−	1.17e+03(2.66e+03)
F21	1.12e+03(8.00e+02)+	1.20e+04(3.35e+04)+	5.88e+02(1.64e+02)+	3.38e+02(1.66e+02)+	2.55e+02(1.15e+02)−	**1.06e+02**(8.27e+01)−	2.90e+02(2.90e+02)
F22	6.41e+01(1.34e+01)−	1.79e+02(3.41e+01)+	5.60e+01(5.48e+01)−	1.20e+02(7.88e+01)=	1.24e+02(6.72e+01)=	**2.35e+01**(3.90e+00)−	1.27e+02(6.51e+01)
F23	2.90e+02(0.00e+00)=	2.90e+02(1.72e−13)=	2.90e+02(1.53e−12)=	2.90e+02(2.10e−13)=	2.90e+02(1.71e−13)=	2.90e+02(1.70e-13)=	**2.90e+02**(1.89e−13)
F24	2.01e+02(4.99e−02)=	2.01e+02(7.72e−02)=	2.01e+02(7.08e−02)=	2.01e+02(5.26e−02)=	2.01e+02(4.09e−02)=	2.01e+02(2.77e-02)=	**2.01e+02**(1.20e−01)
F25	**2.02e+02**(4.91e−01)−	2.10e+02(1.69e+00)+	2.06e+02(2.11e−01)−	2.10e+02(2.42e+00)+	2.08e+02(1.03e+00)=	2.06e+02(2.09e-01)=	2.08e+02(1.48e+00)
F26	1.00e+02(4.24e−02)=	1.00e+02(3.04e−02)=	1.00e+02(6.72e−02)=	1.00e+02(3.73e−02)=	1.00e+02(2.78e−02)=	1.00e+02(1.44e-02)=	**1.00e+02**(3.96e−02)
F27	**3.50e+02**(0.00e+00)−	4.01e+02(1.88e−01)+	4.00e+02(1.33e−01)+	3.96e+02(1.60e+01)+	3.99e+02(1.41e+01)+	3.94e+02(2.38e+01)+	3.73e+02(4.19e+01)
F28	**3.72e+02**(6.97e−01)=	4.34e+02(1.11e+01)=	4.24e+02(4.77e+00)=	4.42e+02(2.43e+01)+	4.17e+02(6.32e+00)=	4.16e+02(2.01e+00)=	4.22e+02(8.57e+00)
F29	1.02e+07(5.13e+06)=	**4.95e+06**(6.11e+06)−	5.84e+06(6.33e+06)−	8.73e+06(7.25e+06)−	8.99e+06(6.19e+06)−	1.28e+07(2.66e+05)+	1.04e+07(4.95e+06)
F30	**5.00e+02**(7.37e+01)−	6.79e+02(1.91e+02)−	5.11e+02(1.54e+02)−	8.07e+02(3.03e+02)+	6.56e+02(1.89e+02)−	6.72e+02(1.33e+02)−	7.55e+02(2.21e+02)
+/=/−	17/5/8	13/11/6	18/5/7	18/4/8	9/12/9	10/8/12	−/−/−
