# Supplementary material for: A novel differential evolution algorithm with multi-population and elites regeneration
Source: PLoS One. 2024 Apr 25;19(4):e0302207. doi: 10.1371/journal.pone.0302207 (PMC11045134; doi:10.1371/journal.pone.0302207)
Supplement: S11 Table — (PDF) [file pone.0302207.s011.pdf]

D=50	**jDE**	**SaJADE**	**CoDE**	**EPSDE**	**SHADE**	**L-SHADE**	**EBJADE**
Fi	**Mean(St.D)**	**Mean(St.D)**	**Mean(St.D)**	**Mean(St.D)**	**Mean(St.D)**	**Mean(St.D)**	**Mean(St.D)**
F1	2.47e+06(1.13e+06)+	3.07e+04(1.93e+04)+	4.27e+05(2.12e+05)+	1.06e+05(5.31e+04)+	3.51e+04(1.80e+04)+	**1.12e+03**(1.58e+03)−	2.13e+04(1.12e+04)
F2	2.98e+00(7.65e+00)+	1.72e−22(2.39e−22)=	4.72e+03(4.35e+03)+	3.32e+03(3.40e+03)+	**1.40e−22**(1.15e−22)=	1.57e-22(1.95e-22)=	2.43e−22(3.04e−22)
F3	1.21e+00(1.97e+00)−	3.62e+03(1.63e+03)+	6.34e−01(9.84e−01)−	3.84e+00(9.27e+00)−	7.83e−26(1.12e−25)−	**1.80e-26**(1.65e-26)−	2.50e+03(2.35e+03)
F4	1.03e+02(2.26e+01)+	**3.92e+00**(1.92e+01)−	8.82e+01(1.87e+01)+	3.54e+01(3.31e+01)+	2.80e+01(4.17e+01)+	8.36e+01(3.33e+01)+	1.37e+01(3.40e+01)
F5	2.08e+01(3.66e−02)+	2.05e+01(2.92e−02)+	2.08e+01(3.87e−02)+	2.08e+01(4.01e−02)+	2.04e+01(2.68e−02)+	2.04e+01(3.61e-02)+	**2.00e+01**(1.72e−03)
F6	3.75e+00(6.94e+00)−	9.50e+00(8.52e+00)−	4.66e+01(1.49e+00)+	1.70e+01(1.33e+01)−	6.78e−01(8.01e−01)−	**2.82e-01**(5.23e-01)−	2.17e+01(7.39e+00)
F7	1.78e−17(4.07e−17)−	2.96e−04(1.45e−03)+	5.91e−03(2.94e−02)+	5.61e−03(7.08e−03)+	2.22e−18(1.55e−17)+	**0.00e+00**(0.00e+00)=	**0.00e+00**(0.00e+00)
F8	4.02e+01(5.02e+00)+	0.00e+00(0.00e+00)−	6.69e−03(6.96e−03)+	1.42e−16(4.82e−16)−	**0.00e+00**(0.00e+00)−	2.71e-08(1.01e-07)+	1.46e−11(1.87e−11)
F9	2.37e+02(1.57e+01)+	7.53e+01(7.28e+00)+	3.15e+02(1.53e+01)+	2.23e+02(1.63e+01)+	6.09e+01(6.53e+00)+	**2.03e+01**(3.13e+00)−	4.52e+01(7.43e+00)
F10	1.05e+03(1.45e+02)+	2.84e+00(6.15e−01)+	8.43e+02(1.32e+02)+	6.34e+02(9.64e+01)+	6.71e+00(1.28e+00)+	**1.62e+00**(9.28e-01)−	2.47e+00(8.64e−01)
F11	9.62e+03(3.82e+02)+	5.70e+03(3.27e+02)+	9.92e+03(3.03e+02)+	9.28e+03(3.46e+02)+	5.08e+03(3.21e+02)+	4.26e+03(2.94e+02)+	**3.94e+03**(3.44e+02)
F12	1.25e+00(1.20e−01)+	5.53e−01(5.01e−02)+	1.33e+00(1.26e−01)+	1.18e+00(1.58e−01)+	4.02e−01(4.27e−02)+	3.56e-01(4.70e-02)+	**2.96e−01**(4.31e−02)
F13	4.27e−01(3.96e−02)+	2.83e−01(3.71e−02)=	6.28e−01(6.54e−02)+	3.92e−01(4.47e−02)+	2.83e−01(3.26e−02)=	**1.68e-01**(2.22e-02)−	2.88e−01(3.69e−02)
F14	3.16e−01(6.62e−02)+	**2.76e−01**(2.78e−02)=	3.55e−01(5.18e−02)+	3.34e−01(1.11e−01)+	2.77e−01(2.63e−02)=	3.35e-01(6.23e-02)+	2.82e−01(2.92e−02)
F15	2.32e+01(1.24e+00)+	9.85e+00(6.96e−01)+	3.56e+01(1.92e+00)+	2.42e+01(1.94e+00)+	9.24e+00(5.62e−01)+	6.50e+00(4.80e-01)+	**6.05e+00**(6.54e−01)
F16	2.07e+01(3.03e−01)+	1.83e+01(3.44e−01)=	2.07e+01(3.19e−01)+	2.03e+01(3.32e−01)+	1.82e+01(3.59e−01)=	1.76e+01(3.93e-01)=	**1.80e+01**(4.10e−01)
F17	6.03e+04(4.57e+04)+	1.83e+03(6.13e+02)−	4.30e+04(2.55e+04)+	1.28e+04(7.22e+03)+	2.17e+03(5.84e+02)=	**1.64e+03**(4.03e+02)−	2.17e+03(5.24e+02)
F18	1.10e+03(1.51e+03)+	**1.03e+02**(2.41e+01)−	1.46e+02(3.88e+01)+	6.16e+02(5.57e+02)+	1.21e+02(3.03e+01)−	1.10e+02(1.69e+01)−	1.32e+02(2.58e+01)
F19	2.58e+01(3.67e+00)+	**6.51e+00**(5.69e+00)−	1.76e+01(2.03e+00)+	2.14e+01(1.50e+01)+	1.88e+01(9.94e+00)+	8.49e+00(2.08e+00)−	1.62e+01(9.34e+00)
F20	2.05e+02(5.28e+01)−	7.22e+03(5.38e+03)+	1.52e+02(5.49e+01)−	1.93e+02(1.26e+02)−	**6.22e+01**(2.30e+01)−	5.14e+01(2.08e+01)−	1.89e+03(4.84e+03)
F21	1.87e+04(1.33e+04)+	2.11e+04(1.42e+05)+	1.25e+04(1.43e+04)+	4.33e+03(2.42e+03)+	1.06e+03(3.46e+02)=	6.98e+02(2.38e+02)−	1.00e+03(3.06e+02)
F22	5.52e+02(1.54e+02)+	5.76e+02(1.31e+02)+	6.08e+02(1.60e+02)+	4.66e+02(1.47e+02)+	3.98e+02(1.22e+02)+	1.19e+02(8.09e+01)−	**3.58e+02**(1.15e+02)
F23	3.27e+02(3.29e−13)=	3.27e+02(3.28e−13)=	3.27e+02(8.70e−10)=	3.27e+02(3.16e−13)=	3.27e+02(3.97e−13)=	3.44e+02(3.56e-13)+	**3.27e+02**(3.26e−13)
F24	**2.03e+02**(1.14e−01)=	2.03e+02(1.62e−01)=	2.04e+02(2.03e−01)=	2.03e+02(1.60e−01)=	2.03e+02(1.49e−01)=	2.85e+02(1.98e-02)+	2.03e+02(1.95e−01)
F25	2.05e+02(1.17e+00)=	2.03e+02(2.95e+00)=	2.05e+02(1.44e+00)=	2.08e+02(2.73e+00)+	**2.03e+02**(2.83e+00)=	2.05e+02(1.40e-01)=	2.05e+02(1.79e+00)
F26	1.00e+02(4.15e−02)=	1.00e+02(2.75e−02)=	1.01e+02(5.72e−02)+	1.00e+02(4.45e−02)=	1.00e+02(2.89e−02)=	1.00e+02(1.94e-02)=	**1.00e+02**(3.51e−02)
F27	4.16e+02(1.20e+00)=	4.11e+02(1.15e+01)=	4.13e+02(1.34e+00)=	4.22e+02(1.13e+01)+	4.19e+02(1.00e+01)+	4.10e+02(6.05e+00)=	**4.10e+02**(9.76e+00)
F28	**4.68e+02**(1.11e+01)=	5.18e+02(1.60e+01)+	4.88e+02(1.19e+01)+	5.83e+02(5.53e+01)+	4.90e+02(1.06e+01)+	9.73e+02(8.35e+01)+	4.71e+02(1.60e+01)
F29	2.95e+03(1.28e+03)+	1.18e+03(2.51e+02)+	2.71e+03(1.20e+03)+	5.76e+06(1.97e+07)+	9.91e+02(7.96e+01)+	9.66e+02(1.93e+01)=	**9.78e+02**(3.96e+01)
F30	5.41e+03(5.35e+02)+	**3.85e+03**(1.02e+03)−	4.93e+03(7.25e+02)+	6.32e+03(1.69e+03)+	4.82e+03(8.98e+02)+	5.95e+03(4.69e+02)+	4.38e+03(1.11e+03)
+/=/−	20/6/4	14/9/7	24/4/2	23/3/4	15/10/5	11/7/12	−/−/−
