# Supplementary material for: A novel differential evolution algorithm with multi-population and elites regeneration
Source: PLoS One. 2024 Apr 25;19(4):e0302207. doi: 10.1371/journal.pone.0302207 (PMC11045134; doi:10.1371/journal.pone.0302207)
Supplement: S12 Table — (PDF) [file pone.0302207.s012.pdf]

D=100	**jDE**	**SaJADE**	**CoDE**	**EPSDE**	**SHADE**	**L-SHADE**	**EBJADE**
Fi	**Mean(St.D)**	**Mean(St.D)**	**Mean(St.D)**	**Mean(St.D)**	**Mean(St.D)**	**Mean(St.D)**	**Mean(St.D)**
F1	1.07e+07(3.44e+06)+	1.92e+05(4.77e+04)=	1.84e+06(5.84e+05)+	4.59e+05(1.19e+05)+	2.63e+05(6.67e+04)+	**1.64e+05**(4.68e+04))−	2.26e+05(7.04e+04)
F2	1.24e+01(3.63e+01)+	**9.34e−22**(1.98e−21)−	1.14e+07(4.00e+07)+	1.59e+04(1.79e+04)+	3.91e−21(1.90e−21)−	2.31e-14(2.84e-14)+	1.13e−18(6.08e−18)
F3	1.66e+01(1.76e+01)−	8.45e+03(2.64e+03)+	1.58e+03(9.61e+02)−	2.02e+01(3.78e+01)−	7.26e−09(3.37e−08)−	**2.98e-15**(5.88e-15))−	4.54e+03(4.77e+03)
F4	1.82e+02(2.61e+01)+	**7.13e+01**(4.72e+01)−	5.08e+02(2.61e+02)−	1.49e+02(3.23e+01)+	1.18e+02(4.23e+01)+	1.74e+02(3.18e+01)+	8.32e+01(3.47e+01)
F5	2.10e+01(3.01e−02)+	2.08e+01(1.45e−02)+	2.12e+01(2.92e−02)+	2.11e+01(3.78e−02)+	2.08e+01(2.24e−02)+	2.07e+01(4.29e-02)+	**2.06e+01**(2.80e−02)
F6	1.83e+01(1.73e+01)−	2.20e+01(1.62e+01)−	1.22e+02(2.74e+00)+	5.14e+01(1.21e+01)−	1.38e+01(3.28e+00)−	**8.92e+00**(2.56e+00))−	7.33e+01(1.75e+01)
F7	6.13e−15(5.04e−15)+	1.87e−03(6.42e−03)+	7.59e−01(3.06e−01)+	2.27e−03(4.92e−03)+	7.87e−04(3.86e−03)+	1.33e-17(3.61e-17)=	**3.55e−17**(6.45e−17)
F8	1.81e+02(7.26e+00)+	9.28e−01(6.79e−01)−	1.92e+02(9.47e+00)+	1.85e+02(8.19e+00)+	1.28e+01(8.56e−01)+	**4.85e-01**(1.74e-01))−	7.13e+00(1.37e+00)
F9	6.57e+02(2.52e+01)+	2.59e+02(1.67e+01)+	9.29e+02(2.75e+01)+	7.15e+02(3.54e+01)+	2.38e+02(1.14e+01)+	**6.59e+01**(1.79e+01))−	1.14e+02(1.38e+01)
F10	6.65e+03(4.63e+02)+	7.12e+02(6.89e+01)+	1.02e+04(4.66e+02)+	6.80e+03(5.61e+02)+	9.16e+02(1.07e+02)+	**1.52e+02**(4.60e+01))−	2.51e+02(8.11e+01)
F11	2.37e+04(5.14e+02)+	1.76e+04(3.59e+02)+	2.65e+04(5.29e+02)+	2.56e+04(7.08e+02)+	1.74e+04(5.55e+02)+	1.39e+04(4.74e+02)+	**1.16e+04**(6.30e+02)
F12	1.86e+00(1.34e−01)+	1.03e+00(4.49e−02)+	2.48e+00(1.97e−01)+	2.35e+00(1.77e−01)+	1.00e+00(4.72e−02)+	6.75e-01(8.93e-02)+	**6.52e−01**(6.86e−02)
F13	5.37e−01(4.83e−02)+	3.46e−01(1.93e−02)=	8.11e−01(5.17e−02)+	4.85e−01(4.03e−02)+	3.49e−01(2.02e−02)=	**2.55e-01**(2.48e-02))−	3.45e−01(2.84e−02)
F14	3.27e−01(2.18e−02)+	2.92e−01(1.32e−02)=	3.89e−01(6.74e−02)+	3.31e−01(2.55e−02)+	2.96e−01(1.75e−02)=	3.48e-01(1.58e-02)=	**2.95e−01**(1.93e−02)
F15	5.90e+01(2.30e+00)+	3.21e+01(2.06e+00)+	4.40e+02(1.78e+02)+	8.20e+01(6.51e+00)+	3.22e+01(1.49e+00)+	2.14e+01(1.32e+00)+	**1.95e+01**(1.27e+00)
F16	4.43e+01(2.77e−01)+	4.18e+01(6.08e−01)+	4.53e+01(4.71e−01)+	4.51e+01(3.05e−01)+	4.17e+01(3.62e−01)+	4.03e+01(5.05e-01)=	**4.00e+01**(5.78e−01)
F17	8.64e+05(3.27e+05)+	1.58e+04(6.67e+03)+	2.61e+05(9.51e+04)+	1.11e+05(3.79e+04)+	1.46e+04(5.12e+03)+	**4.49e+03**(6.20e+02))−	1.30e+04(5.76e+03)
F18	1.50e+03(1.94e+03)+	**2.83e+02**(1.26e+02)−	2.05e+03(1.88e+03)+	9.84e+02(7.36e+02)+	4.65e+02(3.59e+02)+	2.23e+02(1.68e+01))−	4.14e+02(1.65e+02)
F19	8.03e+01(2.19e+01)=	9.16e+01(2.19e+01)+	1.13e+02(1.51e+00)+	1.06e+02(2.42e+01)+	9.46e+01(1.96e+00)+	9.61e+01(2.65e+00)+	**7.94e+01**(2.74e+01)
F20	6.38e+02(9.80e+01)+	8.63e+03(1.65e+04)+	2.34e+03(1.14e+03)+	9.65e+02(3.45e+02)+	3.84e+02(6.41e+01)−	**1.53e+02**(5.57e+01))−	5.81e+02(1.01e+03)
F21	4.07e+05(1.87e+05)+	2.78e+03(6.72e+02)+	1.12e+05(4.63e+04)+	4.91e+04(2.16e+04)+	2.72e+03(8.49e+02)=	2.57e+03(5.77e+02)=	**2.64e+03**(6.90e+02)
F22	2.30e+03(3.20e+02)+	2.10e+03(2.28e+02)+	2.41e+03(1.72e+02)+	1.67e+03(2.92e+02)+	1.74e+03(1.63e+02)+	**1.06e+03**(1.67e+02)	1.18e+03(1.71e+02)
F23	3.48e+02(1.87e−13)=	3.48e+02(7.10e−14)=	3.48e+02(9.51e−04)=	3.48e+02(2.72e−13)=	3.48e+02(3.01e−14)=	3.48e+02(4.96e-14)=	**3.48e+02**(1.86e−13)
F24	3.96e+02(2.99e+00)+	3.85e+02(1.75e+00)+	3.90e+02(4.19e+00)+	4.01e+02(7.34e+00)+	3.86e+02(2.36e+00)+	3.94e+02(3.30e+00)+	**3.79e+02**(3.45e+00)
F25	2.46e+02(5.17e+00)+	2.09e+02(1.55e+01)−	2.39e+02(2.17e+01)+	2.54e+02(1.50e+01)+	2.13e+02(1.91e+01)−	**2.00e+02**(8.43e-14))−	2.22e+02(2.13e+01)
F26	**1.28e+02**(6.63e+01)−	2.00e+02(4.82e−03)=	2.32e+02(7.33e+01)+	2.00e+02(7.43e−03)=	2.00e+02(3.89e−03)=	2.00e+02(1.16e-03)=	2.00e+02(3.59e−03)
F27	4.03e+02(5.71e+01)−	4.51e+02(4.21e+01)−	3.20e+03(8.10e+01)+	1.61e+03(1.38e+02)+	4.49e+02(3.89e+01)−	**3.44e+02**(2.67e+01))−	4.63e+02(6.00e+01)
F28	**1.99e+03**(8.52e+01)−	4.16e+03(6.19e+02)+	3.07e+03(4.54e+02)+	2.81e+03(5.21e+02)+	2.08e+03(1.56e+02)−	2.31e+03(4.04e+01)+	2.21e+03(6.07e+01)
F29	1.93e+03(1.01e+02)+	1.33e+03(1.02e+02)+	1.96e+03(1.51e+02)+	1.33e+03(2.00e+02)+	**8.57e+02**(1.12e+02)−	8.72e+02(1.86e+02)=	8.95e+02(1.78e+02)
F30	**5.85e+03**(1.95e+03)−	6.74e+03(9.02e+02)−	1.15e+04(1.32e+03)+	8.89e+03(1.49e+03)+	7.64e+03(5.89e+02)+	9.35e+03(7.93e+02)+	7.54e+03(1.09e+03)
+/=/−	22/2/6	17/5/8	27/1/2	26/2/2	17/5/8	10/7/13	−/−/−
