# Supplementary material for: A novel differential evolution algorithm with multi-population and elites regeneration
Source: PLoS One. 2024 Apr 25;19(4):e0302207. doi: 10.1371/journal.pone.0302207 (PMC11045134; doi:10.1371/journal.pone.0302207)
Supplement: S13 Table — (PDF) [file pone.0302207.s013.pdf]

D=50	**CMA-ES**	**iCMAES-ILS**	**EBJADE**
Fi	**Mean(St.D)**	**Mean(St.D)**	**Mean(St.D)**
F1	0.00e+00(0.00e+00)	0.00e+00(0.00e+00)	2.13e+04(1.12e+04)
F2	0.00e+00(0.00e+00)	0.00e+00(0.00e+00)	2.43e-22(3.04e-22)
F3	0.00e+00(0.00e+00)	0.00e+00(0.00e+00)	2.50e+03(2.35e+03)
F4	6.25e-01(1.46e+00)	1.03e+01(2.44e+01)	1.37e+01(3.40e+01)
F5	2.01e+01(2.68e-01)	2.00e+01(6.43e-05)	2.00e+01(1.72e-03)
F6	6.84e+01(9.92e+00)	2.24e-04(9.55e-04)	2.17e+01(7.39e+00)
F7	4.83e-04(1.97e-03)	0.00e+00(0.00e+00)	0.00e+00(0.00e+00)
F8	7.18e+02(1.22e+02)	5.75e+00(2.00e+00)	1.46e-11(1.87e-11)
F9	1.15e+03(2.16e+02)	6.36e+00(1.87e+00)	4.52e+01(7.43e+00)
F10	8.58e+03(1.01e+03)	1.60e+02(2.51e+02)	2.47e+00(8.64e-01)
F11	8.07e+03(1.02e+03)	1.12e+02(1.26e+02)	3.94e+03(3.44e+02)
F12	8.79e-02(6.65e-02)	1.02e-02(3.03e-02)	2.96e-01(4.31e-02)
F13	3.63e-01(7.36e-02)	6.59e-02(1.85e-02)	2.88e-01(3.69e-02)
F14	4.60e-01(2.43e-01)	2.42e-01(3.25e-02)	2.82e-01(2.92e-02)
F15	6.10e+00(1.18e+00)	4.56e+00(4.07e-01)	6.05e+00(6.54e-01)
F16	2.38e+01(5.27e-01)	1.99e+01(7.20e-01)	1.80e+01(4.10e-01)
F17	3.14e+03(6.63e+02)	2.12e+03(4.02e+02)	2.17e+03(5.24e+02)
F18	4.75e+02(1.82e+02)	1.71e+02(8.29e+01)	1.32e+02(2.58e+01)
F19	1.94e+01(3.77e+00)	1.44e+01(2.28e+00)	1.62e+01(9.34e+00)
F20	8.12e+02(3.04e+02)	2.92e+02(8.50e+01)	1.89e+03(4.84e+03)
F21	1.77e+03(4.47e+02)	1.54e+03(3.30e+02)	1.00e+03(3.06e+02)
F22	8.01e+02(3.18e+02)	2.01e+02(1.62e+02)	3.58e+02(1.15e+02)
F23	3.37e+02(7.13e-13)	3.44e+02(5.58e-11)	3.27e+02(3.26e-13)
F24	3.82e+02(3.38e+02)	2.62e+02(2.86e+00)	2.03e+02(1.95e-01)
F25	2.00e+02(3.20e-02)	2.05e+02(3.04e-01)	2.05e+02(1.79e+00)
F26	1.14e+02(5.62e+01)	1.00e+02(1.31e-01)	1.00e+02(3.51e-02)
F27	6.18e+02(1.49e+02)	3.10e+02(1.72e+01)	4.10e+02(9.76e+00)
F28	7.42e+03(6.69e+03)	1.24e+03(7.97e+01)	4.71e+02(1.60e+01)
F29	2.20e+02(2.69e+00)	8.05e+02(4.73e+01)	9.78e+02(3.96e+01)
F30	1.42e+03(3.94e+02)	9.48e+03(6.09e+02)	4.38e+03(1.11e+03)
+/=/−	21/0/9	10/5/15	−/−/−
