# Supplementary material for: A novel differential evolution algorithm with multi-population and elites regeneration
Source: PLoS One. 2024 Apr 25;19(4):e0302207. doi: 10.1371/journal.pone.0302207 (PMC11045134; doi:10.1371/journal.pone.0302207)
Supplement: S14 Table — (PDF) [file pone.0302207.s014.pdf]

D=100	**CMA-ES**	**iCMAES-ILS**	**EBJADE**
Fi	**Mean(St.D)**	**Mean(St.D)**	**Mean(St.D)**
F1	0.00e+00(0.00e+00)	0.00e+00(0.00e+00)	2.26e+05(7.04e+04)
F2	0.00e+00(0.00e+00)	0.00e+00(0.00e+00)	1.13e-18(6.08e-18)
F3	0.00e+00(0.00e+00)	0.00e+00(0.00e+00)	4.54e+03(4.77e+03)
F4	7.82e-01(1.60e+00)	1.13e+02(4.73e+01)	8.32e+01(3.47e+01)
F5	2.01e+01(3.12e-01)	2.00e+01(1.67e-05)	2.06e+01(2.80e-02)
F6	1.43e+02(1.20e+01)	7.11e-02(2.23e-01)	7.33e+01(1.75e+01)
F7	3.38e-04(1.71e-03)	0.00e+00(0.00e+00)	3.55e-17(6.45e-17)
F8	1.45e+03(1.58e+02)	1.93e+01(3.05e+00)	7.13e+00(1.37e+00)
F9	2.12e+03(2.72e+02)	2.00e+01(3.30e+00)	1.14e+02(1.38e+01)
F10	1.66e+04(1.40e+03)	9.31e+02(6.50e+02)	2.51e+02(8.11e+01)
F11	1.66e+04(1.50e+03)	1.50e+03(7.95e+02)	1.16e+04(6.30e+02)
F12	4.96e-02(2.64e-02)	6.62e-04(5.94e-04)	6.52e-01(6.86e-02)
F13	5.23e-01(7.43e-02)	2.39e-01(3.17e-02)	3.45e-01(2.84e-02)
F14	2.28e-01(2.39e-02)	1.35e-01(1.62e-02)	2.95e-01(1.93e-02)
F15	1.41e+01(2.27e+00)	9.79e+00(8.36e-01)	1.95e+01(1.27e+00)
F16	4.73e+01(8.27e-01)	4.24e+01(7.67e-01)	4.00e+01(5.78e-01)
F17	5.95e+03(7.88e+02)	5.06e+03(7.46e+02)	1.30e+04(5.76e+03)
F18	1.03e+03(2.88e+02)	5.43e+02(1.68e+02)	4.14e+02(1.65e+02)
F19	6.10e+01(8.92e+00)	8.20e+01(3.35e+01)	7.94e+01(2.74e+01)
F20	1.62e+03(5.24e+02)	6.52e+02(1.24e+02)	5.81e+02(1.01e+03)
F21	3.59e+03(6.05e+02)	3.31e+03(7.39e+02)	2.64e+03(6.90e+02)
F22	1.34e+03(4.24e+02)	5.37e+02(1.78e+02)	1.18e+03(1.71e+02)
F23	3.45e+02(1.88e-12)	3.48e+02(9.27e-09)	3.48e+02(1.86e-13)
F24	6.47e+02(7.07e+02)	3.52e+02(9.48e+00)	3.79e+02(3.45e+00)
F25	2.00e+02(3.83e-03)	2.17e+02(1.85e+00)	2.22e+02(2.13e+01)
F26	1.88e+02(3.24e+01)	1.91e+02(2.71e+01)	2.00e+02(3.59e-03)
F27	1.06e+03(2.17e+02)	3.00e+02(1.92e-01)	4.63e+02(6.00e+01)
F28	2.98e+04(1.20e+04)	2.48e+03(8.67e+01)	2.21e+03(6.07e+01)
F29	2.68e+02(1.06e+01)	1.50e+03(7.18e+02)	8.95e+02(1.78e+02)
F30	2.73e+03(4.68e+02)	8.71e+03(1.31e+03)	7.54e+03(1.09e+03)
+/=/−	15/0/15	11/4/15	−/−/−
