# Supplementary material for: A novel differential evolution algorithm with multi-population and elites regeneration
Source: PLoS One. 2024 Apr 25;19(4):e0302207. doi: 10.1371/journal.pone.0302207 (PMC11045134; doi:10.1371/journal.pone.0302207)
Supplement: S15 Table — (PDF) [file pone.0302207.s015.pdf]

D=50	CMAES	EBJADE	D=100	CMAES	EBJADE
Fi	Time(sec)	time(sec)	Fi	time(sec)	time(sec)
F1	67.852	9.913	F1	458.255	54.209
F2	72.674	7.078	F2	485.094	45.839
F3	65.641	7.393	F3	488.096	47.171
F4	70.812	7.687	F4	468.273	65.342
F5	83.266	8.646	F5	537.857	57.993
F6	103.174	38.722	F6	604.286	164.891
F7	76.936	5.677	F7	511.633	49.665
F8	76.294	4.026	F8	470.624	14.582
F9	80.383	9.678	F9	514.251	50.451
F10	67.267	5.497	F10	437.627	21.957
F11	70.088	9.924	F11	493.381	55.045
F12	122.024	39.334	F12	611.486	186.947
F13	90.731	6.852	F13	496.641	43.404
F14	79.226	7.047	F14	478.855	44.039
F15	69.782	9.209	F15	463.016	50.152
F16	64.154	9.427	F16	435.465	50.671
F17	64.755	10.413	F17	448.869	53.354
F18	56.226	8.575	F18	374.449	45.933
F19	65.931	16.586	F19	467.013	80.601
F20	66.923	7.547	F20	449.048	52.886
F21	67.224	8.834	F21	444.608	51.042
F22	76.352	11.767	F22	465.444	65.092
F23	122.913	50.837	F23	823.495	372.684
F24	90.958	30.066	F24	622.511	191.715
F25	92.237	34.856	F25	694.824	236.201
F26	150.124	81.709	F26	951.189	513.716
F27	160.722	83.646	F27	945.235	511.631
F28	125.283	53.646	F28	838.875	425.949
F29	91.799	39.174	F29	711.282	259.965
F30	91.847	39.802	F30	688.331	241.438
